# Food Salience and the Nutrition Transition in Kiribati: Urban–Rural and Socioeconomic Patterning of Food Preferences

**DOI:** 10.3390/foods15193499

**Published:** 2026-09-29

**Authors:** Zhen Ming Jeremy Ng, John Paul Cauchi

**Affiliations:** Faculty of Medicine and Dentistry, Queen Mary University of London, Malta Campus, Triq l-Arcisqof Pietru Pace, VCT 2520 Victoria, Gozo, Malta; jeremyngzm@outlook.com

**Keywords:** Kiribati, food choice, cultural salience, food preferences, nutrition transition, urbanisation, pacific island countries and territories, food policy

## Abstract

Kiribati is undergoing a nutrition transition as imported foods increasingly compete with traditional staples, yet the cultural patterning of food preferences remains poorly characterised. We analysed 2019 cross-sectional household survey data from 399 adults primarily responsible for food preparation. Three timed freelists captured favourite foods, traditional foods, and the three foods eaten most often. Smith’s salience, cultural consensus analysis, hierarchical clustering, and multivariable linear regression characterised food preferences and socioeconomic correlates of the proportions of imported, processed and traditional items in favourite-food lists. Fish, breadfruit, and coconut formed a cross-domain core of salient foods, while imported rice ranked first among frequently consumed foods and was salient across all three domains in rural participants. Urban respondents showed stronger cultural consensus than rural respondents (6.57–7.69 vs. 3.60–5.65). Spending > 75% of household income on food was associated with greater imported (β = 0.13, *p* ≤ 0.05) and processed (β = 0.16, *p* ≤ 0.01) food preferences. Urban residence, refrigerator ownership, and larger household size were associated with a lower proportion of traditional items in favourite-food lists. Freelisting revealed that culturally valued local foods persist despite widespread import dependence, suggesting that observed consumption does not fully represent collective food preference. Food-choice interventions are therefore worth testing as ways to make already-valued local foods more affordable, convenient, and accessible.

## 1. Introduction

The nutrition transition describes a shift from traditional diets, rich in complex carbohydrates, fibres and unsaturated fats, towards Westernised diets high in refined carbohydrates, sugar, salt and saturated fats [1]. These imported, ultra-processed foods are often affordable, convenient, hyper-palatable, and heavily marketed, reinforcing dependency and accelerating dietary change [2,3]. Across Pacific Island Countries and Territories (PICTs), this transition has contributed to a high burden of obesity, diabetes, hypertension, and other non-communicable diseases (NCDs) [4,5]. Kiribati provides an informative setting to explore how these processes have influenced food choices, as despite its remoteness from major shipping routes, the nation increasingly relies on imported staples.

**Figure 1:**
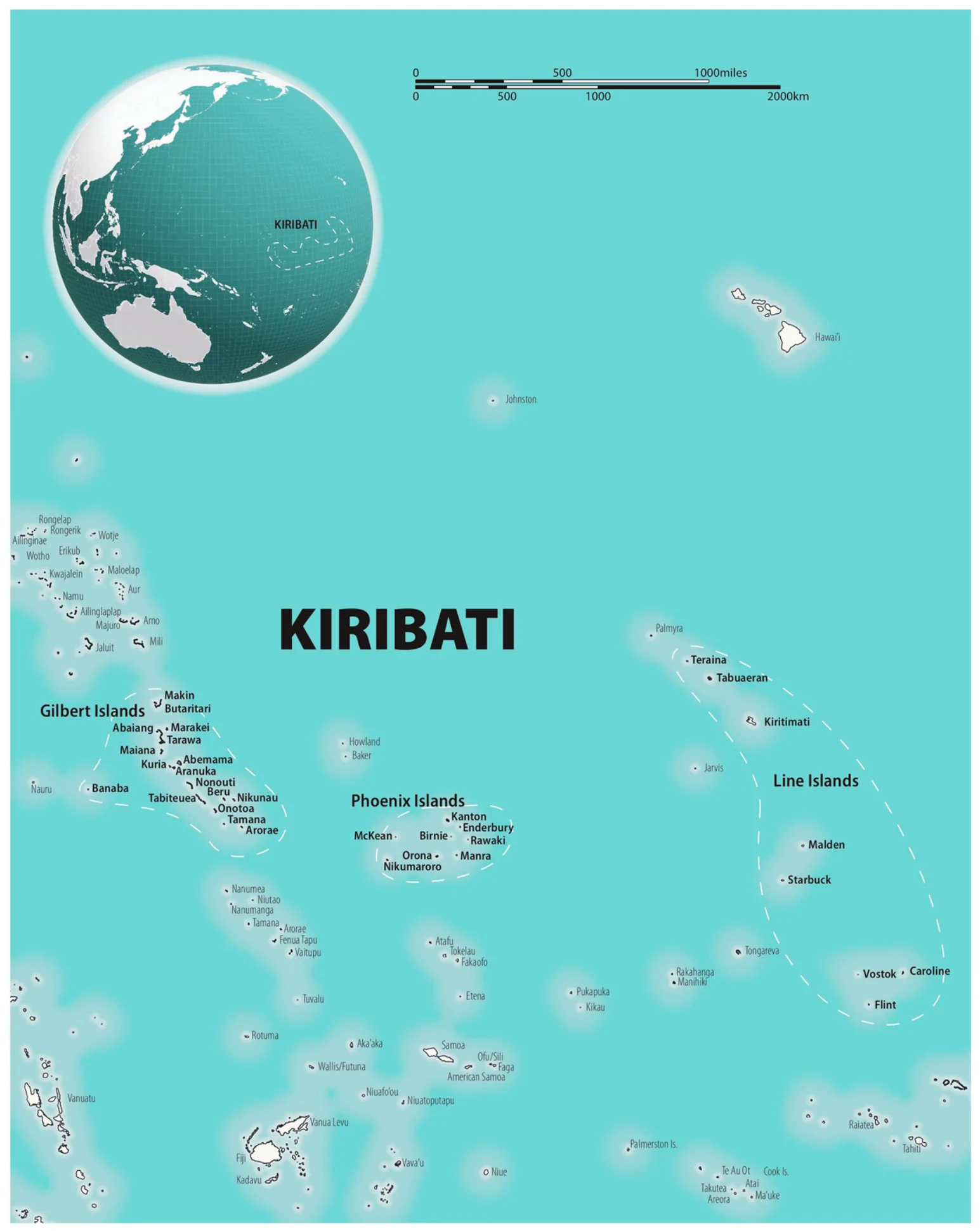
Location of Kiribati in the Pacific Ocean. Source: Mark Gunning ©.

The Republic of Kiribati comprises 32 low-lying atolls and one raised limestone island across 3.5 million km^2^ of ocean (Figure 1). More than half the population now reside in South Tarawa, where high population density, land scarcity and infrastructure constraints restrict household food production and increase dependence on market supply [6,7,8]. Atoll agriculture is further constrained by calcareous soils, freshwater limitations, and climate-related pressures on food and water systems [9,10,11]. Imported foods now account for a large share of dietary energy, with rice alone comprising a substantial proportion of energy intake [12]. At the same time, traditional staples such as breadfruit, fish, coconut, babai (swamp taro), pandanus, and other locally sourced foods remain historically and culturally important [13,14]. The nutrition transition therefore involves changes in food supply, acquisition, preparation and consumption within changing social and economic conditions.

For this study, “traditional” or “local foods” refer to locally sourced staples such as breadfruit, babai, coconut, and fresh fish. “Imported” foods refer to commodities not historically produced or sourced in Kiribati, including rice, sugar, flour and processed meats. Some items, notably chicken and eggs, are both produced domestically and imported, and the origin of any single mention cannot be determined from a freelist response; Supplementary File S5 sets out how each item was handled. Due to long shipping times to Kiribati, eggs are assumed to be locally sourced. This reflects historical origins and current production and distribution pathways, as well as the way local i-Kiribati refer to their foods.

Within this mixed food system, household food provisioning draws on purchased staples, locally harvested foods, and foods exchanged through family and community networks. Previous studies have characterised the nutrition transition in Kiribati through dietary intake, food-security and qualitative data, documenting increasing dependence on imported foods alongside substantial household and ecological constraints [15,16,17]. Eme et al. [15] studied 161 households in South Tarawa using dietary recall and diversity measures, whereas Kodish et al. [16] combined freelisting, interviews and observation to investigate infant and young-child feeding in urban and rural settings. Qualitative data obtained by Cauchi et al. also describes taste, familiarity, and convenience as influences on food choice [6,15,18]. Together, these influences mean that observed food consumption may reflect the opportunities and constraints of the food environment as well as underlying preference. Understanding the local nutrition transition therefore requires consideration of both what people eat and what they value. However, the foods preferred by adults responsible for household food preparation, and how these preferences relate to concepts of tradition and reported frequent consumption, remain poorly characterised.

Cultural salience captures the foods that are most frequently and prominently recalled within a specified domain [19]. This can be achieved through freelisting, in which respondents spontaneously list foods in response to a prompt, allowing commonly and prominently recalled items to be identified [20,21]. Comparing favourite, traditional and most frequently eaten foods freelists can therefore show whether imported staples are becoming culturally embedded or coexist with enduring preferences for local foods, and whether these patterns differ between urban and rural food environments. Identifying highly salient local foods can also provide culturally meaningful candidates for community discussion and intervention development, while differences between preference and reported consumption can highlight food-system factors such as affordability, availability, storage and convenience for further investigation. These findings can therefore help inform interventions aimed at making culturally valued foods more accessible and practical within the changing Kiribati food system.

Therefore, this study aimed to (1) describe the frequency and cultural salience of foods identified as favourite, traditional, and top-three eaten most often; (2) compare shared cultural consensus structures for favourite foods between urban and rural respondents, with corresponding analyses of the other two domains; (3) examine how food repertoires cluster by locality, food expenditure, local harvesting, and education; and (4) estimate associations of urban–rural locality, education attainment, employment status, reported food expenditure share, and other household characteristics with the proportions of imported, processed and traditional food items in culturally salient favourite foods.

## 2. Materials and Methods

### 2.1. Study Design and Setting

This study is a secondary analysis of a larger cross-sectional household survey conducted in Kiribati in 2019, before the COVID-19 pandemic. The parent survey included 402 households, with 399 respondents completing the freelisting questions. Data collection covered South Tarawa (including Betio), North Tarawa, and Butaritari in the Gilbert Islands.

### 2.2. Sampling and Participants

Sampling was based on enumeration areas (EAs) provided by the Kiribati National Statistics Office (KNSO) in collaboration with the Secretariat of the Pacific Community (SPC). The country is divided into 391 EAs. EAs were randomly selected using Microsoft Excel’s RAND function, and 10 households per EA were surveyed using a systematic “s-bend” approach in the absence of a formal address system. Within each household, the primary person responsible for food preparation was invited to participate. Power calculations indicated that a minimum sample size of 100 participants would be sufficient to detect moderate differences in salience between subgroups (α = 0.05, power = 0.80). The final sample of 402 exceeded this threshold, supporting subgroup comparisons. While efforts were made to include a range of locations, 85% of the sample was drawn from urban areas, reflecting population distribution but also introducing potential sampling bias that may limit generalisability to rural communities. Details of sampling and power calculations are provided in Supplementary File S1.

### 2.3. Freelisting Data Collection

Five trained research assistants fluent in both English and i-Kiribati conducted interviews in the respondent’s preferred language. Responses were anonymised, manually cleaned, and translated to standardised English food-item lists.

Participants were given one minute for each open freelisting prompt, with no food examples or suggestions provided by interviewers: (1) ‘What are your favourite foods?’; (2) ‘What are your traditional foods?’; and (3) ‘What three foods do you eat most often?’. These questions were based both on the CRiSTAL Food Security Manual [22] and similar investigations [23,24]. The survey items are reproduced in Supplementary File S2.

Importantly, no definition of “traditional” was offered, and interviewers were instructed not to clarify the term if asked. The domain was deliberately left to the respondent, since the purpose of freelisting is to recover the participants’ own category rather than to test recall against a definition supplied by the researcher.

### 2.4. Salience Analysis

Freelisting data were analysed using the AnthroTools package (version 2.01) in R [25]. Smith’s S combines the frequency with which an item is mentioned and its rank within each respondent’s list [19].

Salience was calculated in three steps. First, each item in a respondent’s list was scored by its position: in a list of L items, the item in position k received a score of (L − k + 1)/L, so the first item scored 1 and the last scored 1/L. Where a respondent named the same food twice, the higher score was kept. Second, each respondent’s scores were rescaled to sum to one, so that every respondent contributed equally regardless of how many foods they listed. Third, Smith’s S for a food was taken as the mean of these rescaled scores across all 399 respondents; it was zero for those who did not mention it. Smith’s S therefore ranges from 0 to 1, and a higher value means a food was both named by more people and named earlier in their lists.

Within each domain, foods were ranked from highest to lowest Smith’s S and plotted against their rank. This is known as a scree plot, analogous with the loose rock that gathers at the foot of a cliff: the curve falls steeply across the first few foods and then flattens into a long tail of items that few respondents mentioned, or mentioned only at the end of their lists. The point at which the curve flattens, the elbow, was taken as the boundary of the culturally salient set for that domain. This approach follows practical guidance for freelisting analysis [20,21]. Identifying the elbow involves analyst judgement; it was applied consistently across all three domains, and the complete ranked lists and scree plots are given in Supplementary Files S3 and S4 so that readers can assess the cut-points themselves.

### 2.5. Cultural Consensus Analysis

Cultural consensus analysis tests whether a group of respondents share a single body of knowledge about a domain, and estimates what that shared knowledge is [21]. Rather than assuming in advance which responses are culturally correct, it infers them from the pattern of agreement between respondents: the more two people’s responses coincide, the more likely both are drawing on the same shared model. Respondents whose responses align closely with those of the group are treated as more knowledgeable about the domain, and are weighted more heavily in the estimate of the shared model.

Cultural consensus analysis was conducted with AnthroTools for the full sample and urban and rural subgroups. Agreement between every pair of respondents was calculated from participant-by-item response matrices, and the resulting respondent-by-respondent agreement matrix was decomposed to derive eigenvalues representing shared cultural knowledge. A first-to-second eigenvalue ratio of at least 3:1 was interpreted as evidence of a shared cultural model [21]. Individual cultural competence is each respondent’s estimated agreement with that shared model, on a scale from 0 to 1. Mean and range of competence scores were reported, together with the number of estimates outside the expected 0 to 1 range. Residual model fit was assessed using the root-mean-square residual (RMSR) between observed pairwise agreement and agreement predicted by the consensus model. Ratios, competence scores and residual fit were compared descriptively across locality and freelisting domains.

Because the traditional food prompt was left open (Section 2.3), consensus analysis also serves as an empirical check on whether respondents were drawing on a shared underlying concept rather than their own personal definitions.

### 2.6. Hierarchical Clustering

Hierarchical clustering was used to visualise items based on how frequently and similarly they are mentioned [26]. Responses were processed into rank-based matrices, where each row represented a food item and each column a respondent. Average absolute rank differences (city-block distance) were calculated between food items, followed by agglomerative hierarchical clustering using average linkage. Only items mentioned by at least 10% of respondents in each group were retained. Dendrograms were generated using the factoextra package (version 1.0.7) in R [27]. Four subgroup characteristics were examined: (1) locality (urban vs. rural), (2) food expenditure (≤50% vs. >50% of income), (3) food harvesting (yes vs. no), and (4) education level (low vs. high). Clusters were visualised using coloured dendrograms, with rectangular borders representing k = 4 item groupings.

### 2.7. Multivariable Regression Analysis

For each respondent’s list, the favourite food freelist was coded to generate three dependent variables: the proportion of listed foods that were (1) imported, (2) processed, or (3) traditional (defined as both local and unprocessed). Three separate multiple linear regression models were fitted, one for each variable. Predictors were selected based on relevance to food access and cultural practices, including age category; education; employment category; proportion of household income spent on food (<25%, 25–50%, 51–75%, >75%); urban versus rural locality; household size; refrigerator ownership; freezer use; harvesting of local food; sex; Tarawa versus outer-island residence; floor area per capita; and floor area per household. These variables are household-level proxies for food access. Food prices, retail availability, and the range of foods obtainable locally were not recorded in the parent survey and so were not available for analysis. All continuous independent variables (household size, floor area per capita, and floor area per household) were standardised to a mean of zero and a standard deviation of one prior to model fitting, so that their beta coefficients would be directly comparable; all remaining predictors were categorical and dummy coded against a reference level. To aid interpretation, these were visualised in a comparative heatmap (Section 3.5).

## 3. Results

### 3.1. Participant Characteristics

A total of 399 participants (99%) completed the freelisting questions. Respondents were predominantly female (270/399, 68%) and from urban areas (339/399, 85%). Most were between 31 and 50 years of age (215/399, 54%). Education ranged from no formal schooling to university-level. Median household size was 7 (IQR 5–9). Table 1 lists the villages assigned to the urban and rural categories, and Table 2 summarises participant characteristics.

Rural respondents obtained local food directly, through harvesting (88.3% against 55.5% urban) and fishing (87.9% against 42.3%), while urban respondents bought it, at the fish market (65.5% against 20.7%) and generally (82.0% against 48.3%). Food preservation practices reflected differences in appliance access: urban respondents reported greater use of refrigeration (63.7% versus 11.7%) and higher refrigerator ownership (50.6% versus 11.7%) than rural respondents. Conversely, salting was more common among rural respondents (86.7%) than urban respondents (34.8%). Urban respondents shared and received local food more often, yet were also more likely to report eating a less-preferred food because of ease or cost (51.1% against 22.0%). Rural counts are small, at roughly 58 to 60 per question, so those percentages carry wide uncertainty. These results are described in Table 3 below.

### 3.2. Overall Food Salience Across Cultural Domains

Across all domains, Smith’s S declined sharply after the highest-ranking foods (Supplementary File S3), indicating that a relatively small set of items dominated spontaneous food recall. In the favourite food domain, fish (0.204), rice (0.148), breadfruit (0.090), chicken (0.063), babai (0.036), and coconut (0.036) comprised the salient set. In the traditional food domain, breadfruit (0.174), fish (0.162), coconut (0.104), babai (0.096), pumpkin (0.063), pandanus (0.050), and banana (0.042) comprised the salient set. The three foods eaten most often, rice (0.363), fish (0.354), breadfruit (0.074), chicken (0.068), and coconut (0.031), comprised the salient set.

**Figure 2:**
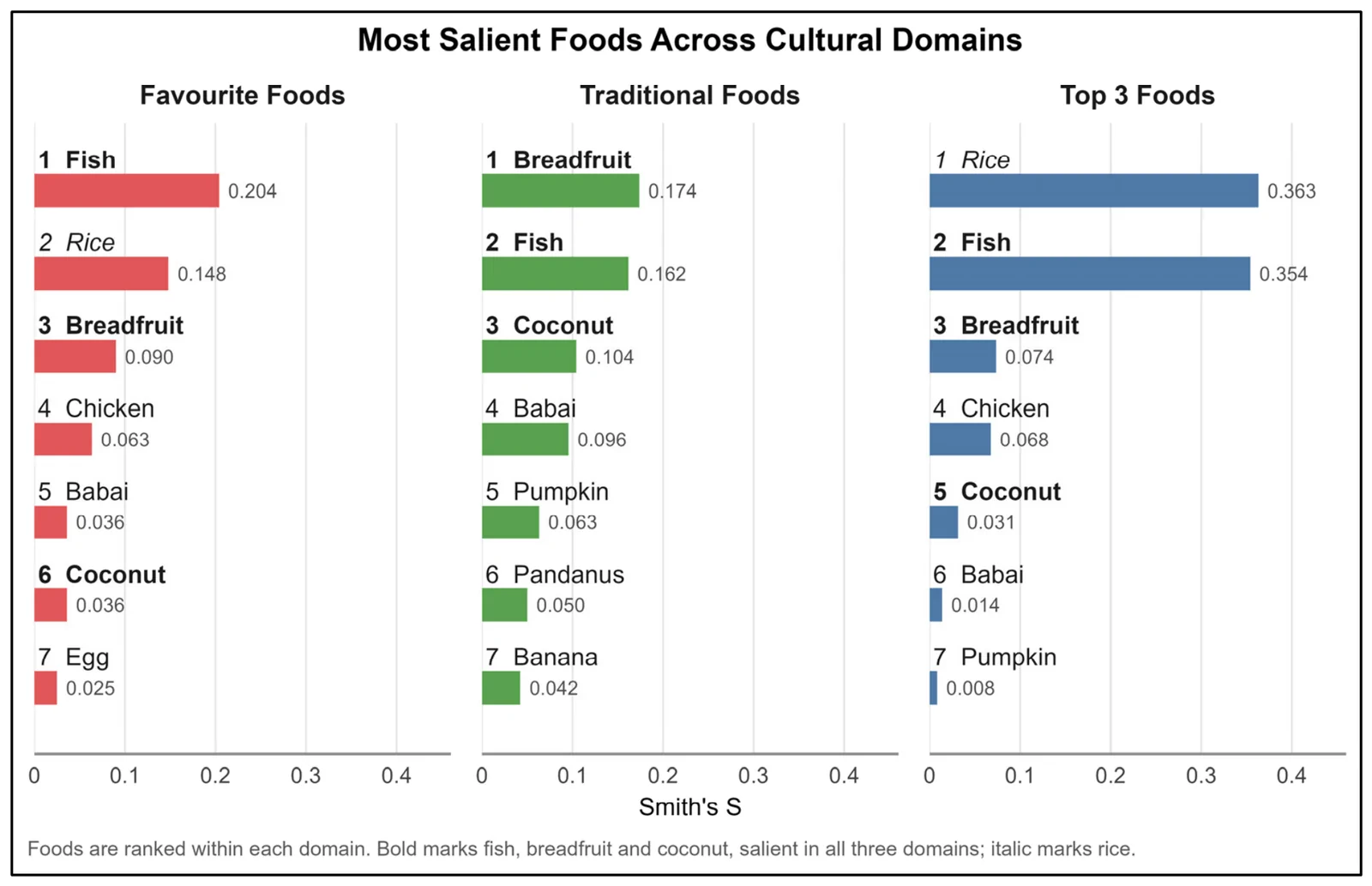
Seven highest Smith’s S scoring food items across the three freelisting domains.

Fish and breadfruit consistently ranked in the top-three items across all three domains, with coconut also being salient. These foods form a cross-domain core of culturally prominent foods. Rice and chicken were salient in favourite and frequently consumed lists but were absent from traditional foods. Breadfruit dominated traditional foods, but salience across this domain was more evenly distributed across items like coconut, babai, pumpkin, pandanus, and banana. Figure 2 displays the seven highest Smith’s S scoring foods across the three domains.

### 3.3. Urban–Rural Differences in Food Salience and Consensus

**Figure 3:**
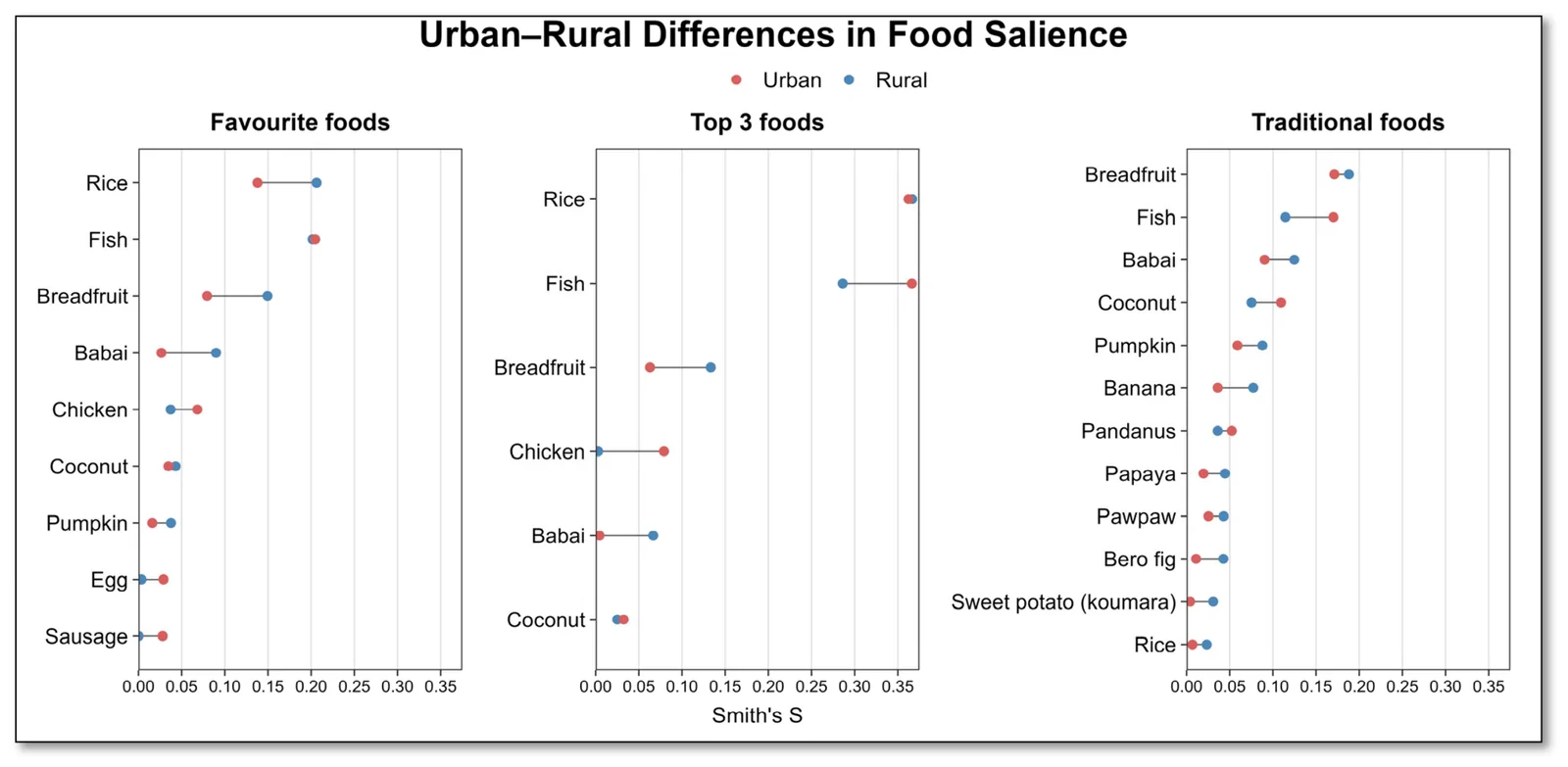
Urban–rural differences in Smith’s S salience across favourite, top-three frequently eaten, and traditional food domains. Foods shown were salient in at least one locality within each domain. Paired points display the corresponding Smith’s S in both groups.

Urban–rural comparison showed both a shared core and marked differences in the relative salience of specific foods (Figure 3; Supplementary File S4). Rural participants had higher salience for rice in favourite foods (0.206 vs. 0.138), breadfruit in favourite foods (0.149 vs. 0.079) and the top-three domain (0.133 vs. 0.063), and babai across all three domains. Urban participants showed higher salience for chicken in the top-three domain (0.079 vs. 0.003), and egg (0.029 vs. 0.003) and sausage (0.028 vs. 0.000) were more prominent among urban favourite foods. Traditional food repertoires were broader among rural respondents, with higher Smith’s S values for babai (0.125 vs. 0.090), pumpkin (0.088 vs. 0.059), banana (0.077 vs. 0.036), papaya (0.045 vs. 0.020), bero fig (0.043 vs. 0.011), and sweet potato (0.031 vs. 0.004). Imported rice was retained as salient in the rural traditional foods, so rice was salient across all three rural domains. Urban participants listed slightly more items on average than rural participants in favourite foods (6.5 vs. 5.6) and traditional foods (6.4 vs. 5.8), but the rural salient set contained a wider range of traditional plant foods. Cultural consensus analysis supported a dominant shared response pattern across all three food domains in the full sample, with first-to-second factor eigenvalue-ratios of 8.05 for favourite foods, 7.07 for traditional foods and 6.82 for top-three foods. Mean cultural competence was high across domains (0.91–0.96), with no negative competence estimates or estimates exceeding 1. Subgroup analyses also met the conventional ≥3:1 criterion in both urban and rural respondents. Urban groups showed higher factor ratios and mean competence than rural groups across all three domains, while residual disagreement was greater in the rural traditional-food and top-three-food models (Table 4).

### 3.4. Hierarchical Clustering of Food Repertoires

**Figure 4:**
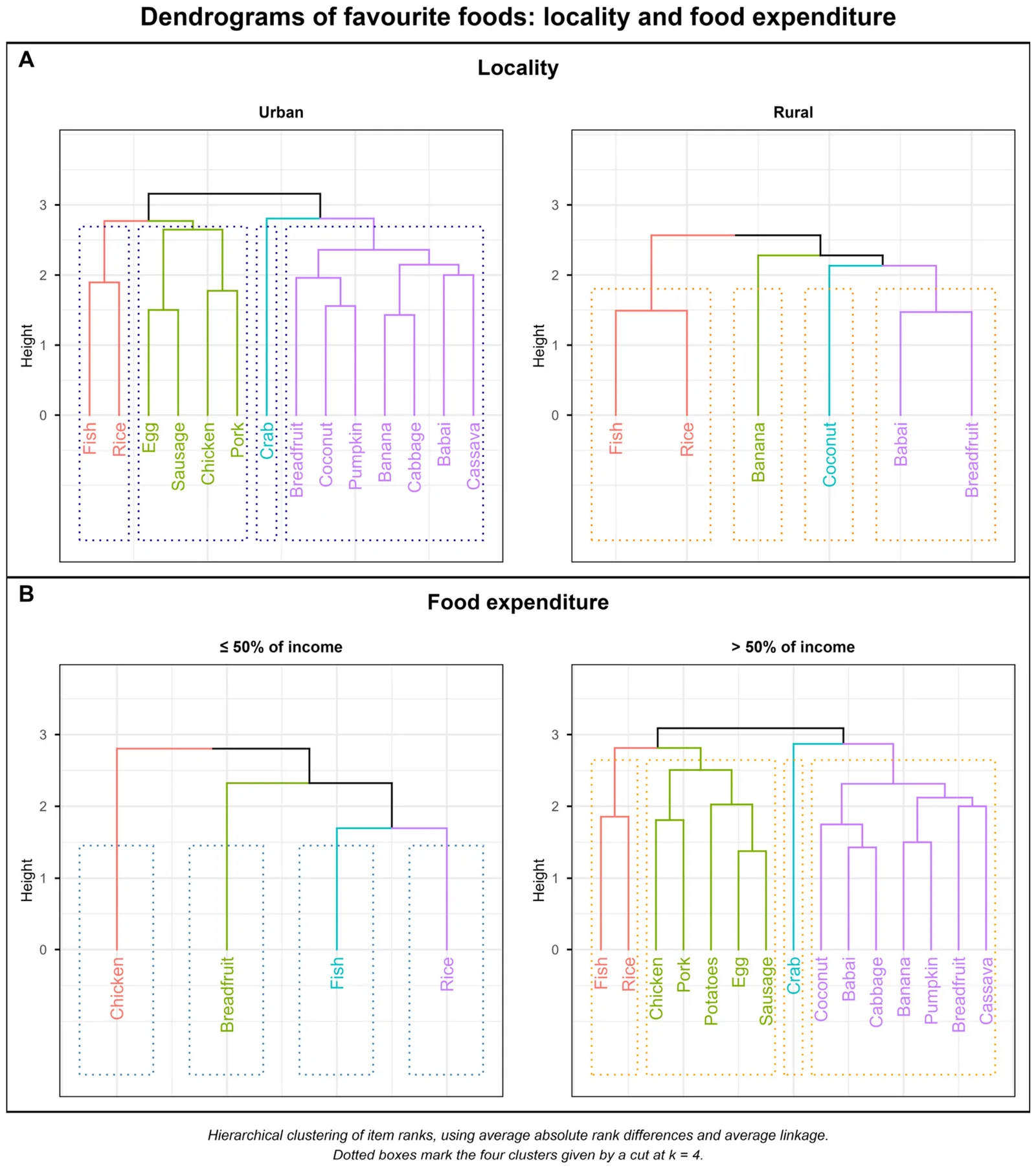

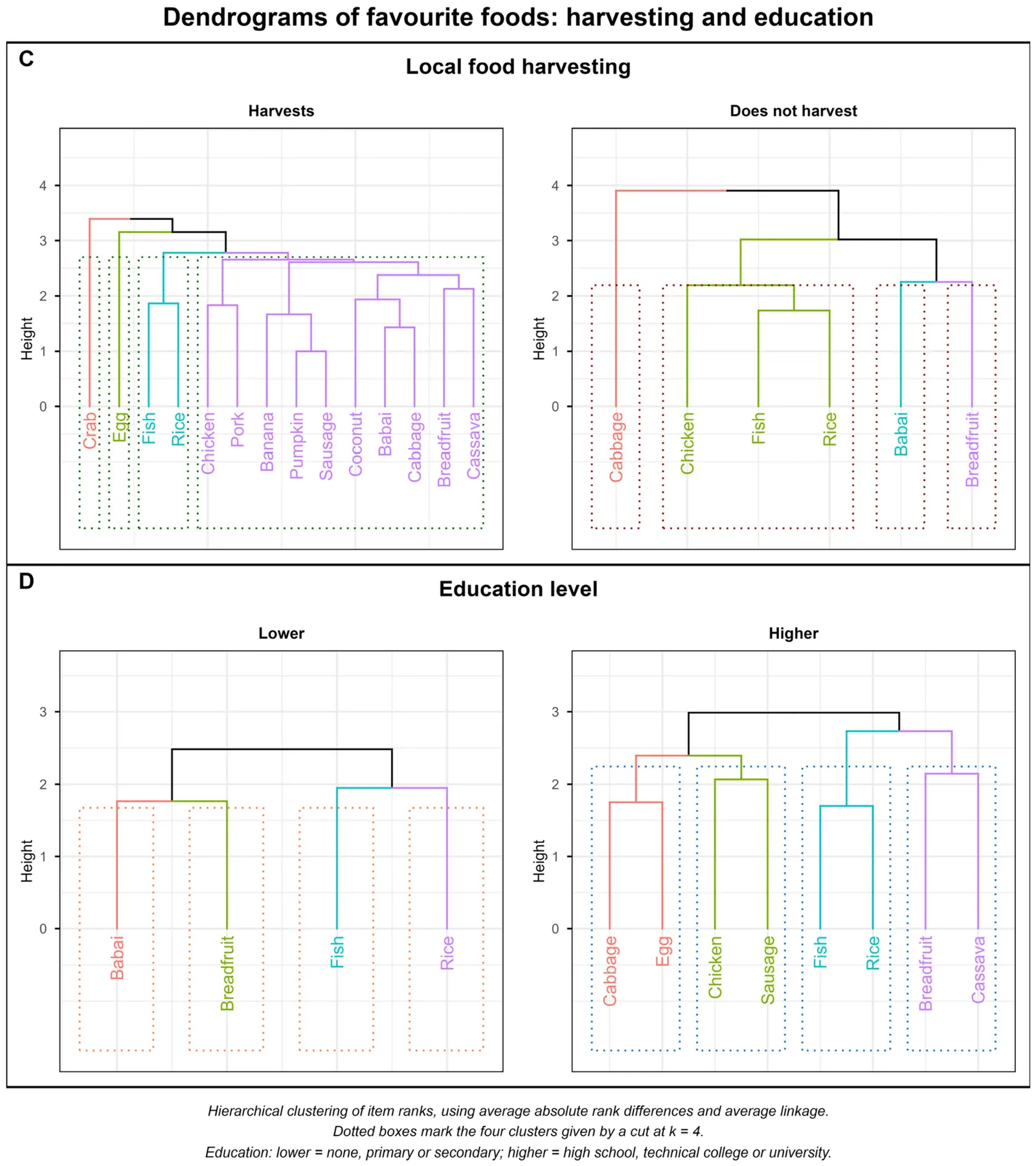
Hierarchical clustering of favourite food freelists based on average rank differences. The height at which items join indicates how often they were mentioned together: lower joins mean items were frequently listed by the same people, while higher joins suggest they were mentioned by different individuals. (**A**) Urban versus rural; (**B**) food expenditure ≤ 50% versus >50% of household income; (**C**) households harvesting food locally versus not; (**D**) lower versus higher education. Dotted rectangles denote k = 4 visual groupings.

The dendrograms further illustrated heterogeneity in food repertoires (Figure 4). Rice and fish frequently occupied closely related branches across subgroup analyses. Urban favourite food repertoires included tightly related clusters containing processed or market-supplied foods such as egg, sausage, chicken, and pork, whereas rural repertoires contained fewer retained items and were more strongly anchored around local staples. Participants spending > 50% of household income on food displayed a wider and more fragmented repertoire containing more imported and processed items than those spending ≤ 50%. Higher-education respondents also showed broader clustering, while lower-education respondents retained fewer items. Households harvesting food locally displayed a mixed repertoire containing both imported and local items.

### 3.5. Socioeconomic and Household Correlates of Favourite Food Freelist Composition

**Figure 5:**
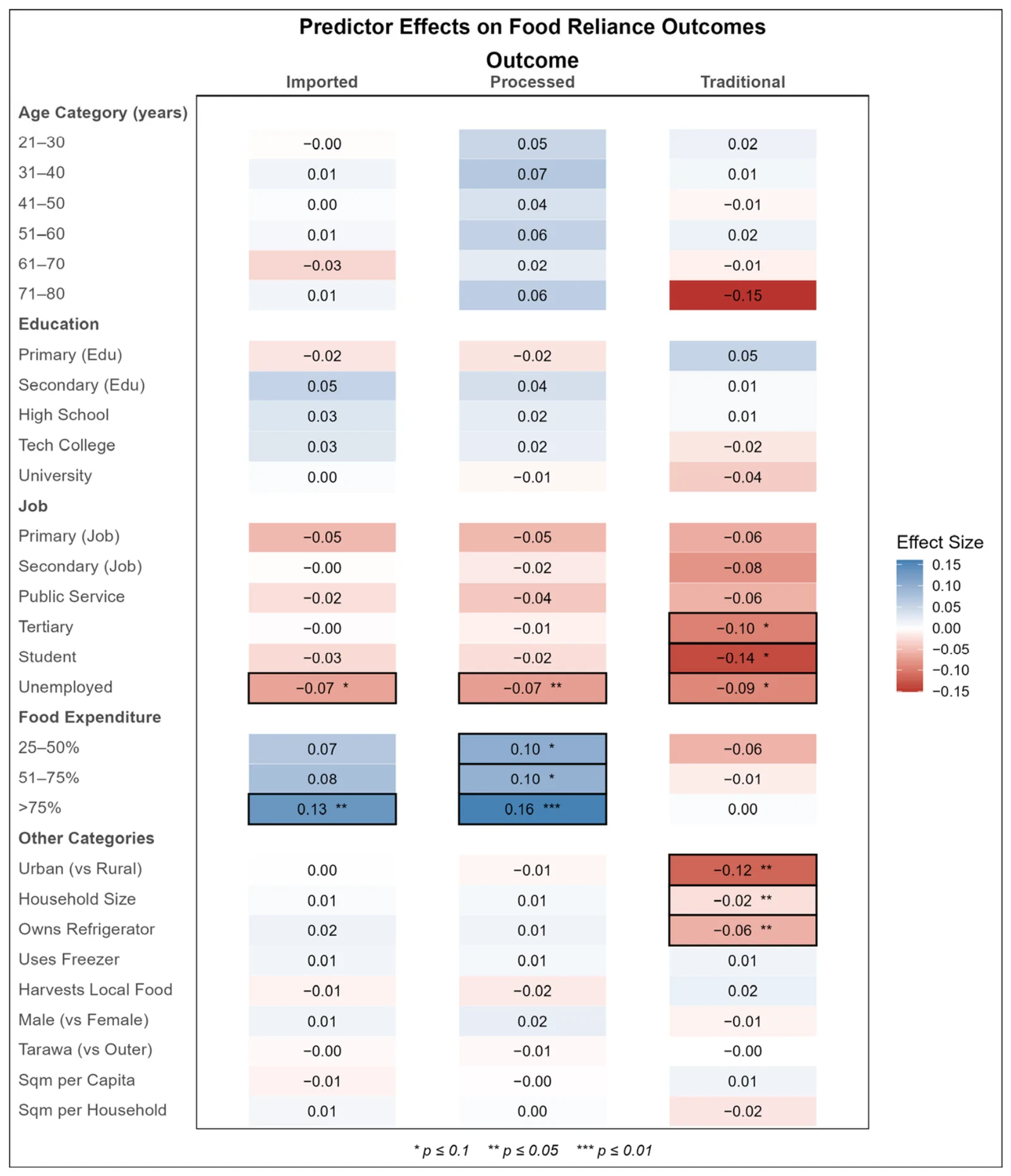
Regression coefficients for imported, processed, and traditional food proportion in favourite food freelists. Predictors are grouped by age, education, job, food expenditure, and other household characteristics.

The multivariable models identified several socioeconomic and household correlates of the composition of favourite food freelists (Figure 5). Spending > 75% of household income on food was positively associated with the proportion of imported foods (β = 0.13, *p* ≤ 0.05) and processed foods (β = 0.16, *p* ≤ 0.01). The 25–50% and 51–75% expenditure categories each showed marginal positive associations with greater processed-food proportions (β = 0.10, *p* ≤ 0.10). Unemployment was associated with a lower processed-food proportion (β = −0.07, *p* ≤ 0.05) and showed marginal inverse associations with imported (β = −0.07, *p* ≤ 0.10) and traditional (β = −0.09, *p* ≤ 0.10) proportions. Urban residence (β = −0.12, *p* ≤ 0.05), refrigerator ownership (β = −0.06, *p* ≤ 0.05), and larger household size (β = −0.02, *p* ≤ 0.05) were inversely associated with the proportion of traditional foods listed. Tertiary employment (β = −0.10, *p* ≤ 0.10) and student status (β = −0.14, *p* ≤ 0.10) also showed marginal inverse associations.

## 4. Discussion

### 4.1. Core Findings

Kiribati’s nutrition transition is characterised by coexistence between enduring local-food preferences and the incorporation of imported staples into everyday food culture. Fish, breadfruit and coconut remained salient across favourite, traditional and frequently eaten domains, indicating that a core of culturally important local foods persists despite substantial import dependence. At the same time, rice was among the most prominent foods in the dataset and had become sufficiently incorporated to enter the salient traditional food repertoire of rural respondents. Together, these findings extend previous work describing what households consume by showing how specific foods are collectively valued and culturally organised [6,15,16,17]. By combining freelisting with household-level and socioeconomic subgroup analyses, this study captures both the foods that communities collectively value and the food-system circumstances within which those preferences are expressed. The clearest expression of this pattern was the contrast between urban and rural food repertoires.

### 4.2. Urban–Rural Food Preference Landscapes

Urban and rural respondents shared many core foods, yet differed in repertoire structure and cultural consensus. Urban respondents showed stronger cultural consensus across all three freelisting domains, suggesting a more standardised set of shared food preferences. Their lists also gave greater prominence to chicken, egg, sausage, and other market-supplied foods.

The stronger urban consensus observed here may have several explanations. Previous research in Kiribati has described more standardised retail supplies and constraints related to food storage, availability, and time pressures in urban settings, which could contribute to the pattern observed here [6,17,28,29]. In contrast, rural participants retained a wider salient repertoire of babai, pumpkin, banana, papaya, Bero fig, sweet potato, and other traditional foods. A possible explanation could be stronger access to subsistence food production and local ecological knowledge.

Rice complicates a simple imported-versus-traditional dichotomy. It was more salient in rural than urban favourite food lists, remained dominant in the rural top-three domain, and crossed the rural traditional food salience threshold. Rice is a long-established imported staple with clear advantages in storage, transport, preparation predictability, and year-round availability as opposed to local staples like breadfruit. The historical origins of rice becoming a staple food in Kiribati dates to the late 19th and early 20th century, when many workers from the Gilbert Islands worked on the phosphate mines in Banaba. Here, rice was given to workers as an easily cookable, transferable and stored food item, and it quickly became a preferred staple food, despite the fact that it cannot be grown on Kiribati [30]. This also reflects deeper cultural assimilation of rice into traditional diets, as observed in a previous study, wherein elderly rural i-Kiribati were observed mixing rice with traditional foods when preparing meals [6]. Rice could enter the rural traditional food repertoire precisely because the prompt did not constrain what counted as traditional; a researcher-supplied definition for it would have excluded it a priori and the result would have been unobservable. The mention of rice by many participants as a traditional food shows how intrinsically tied with i-Kiribati diets it has become, despite it not being grown in Kiribati.

Fish showed a different pattern as it remained highly salient in urban respondents. Previous research has documented constraints on access to locally produced and fresh foods in South Tarawa [6,16,31]. Its high salience may be due to enduring cultural attachment and scarcity-related value, often observed by the author of this manuscript and visible on South Tarawa, where fish from outer islands are sold along the main road and are taken up at a fast rate for consumption. Observational work in Kiribati similarly emphasises that food preferences are embedded in social meaning as well as practical availability [32].

### 4.3. Socioeconomic Correlates and Possible Explanations

The regression findings show that higher food-expenditure burden was associated with a greater proportion of imported and processed foods in favourite food lists. Urban residence, refrigerator ownership, and larger household size were also associated with lower proportions of traditional foods. Note that these are associations between household characteristics and the composition of freelists. The explanations considered below build on previous research.

A clarification regarding “unemployment” status is necessary. Unemployment here usually indicates the lack of a regular, employed position, which in turn indicates a reliance on subsistence. This reduces reliance on a cash economy.

For traditional foods, urban residence, refrigerator ownership, and larger household size were all significantly associated with a lower proportion of traditional items in favourite-food lists. Note that refrigeration here reflects access to electricity, which is notably lacking in rural areas.

Previous qualitative research identified availability, storage and preparation convenience as factors that may influence food choice, providing plausible explanations for these associations [6,18]. Meal source may also be relevant, as national household expenditure evidence indicates that 64% of households reported having lunch away from home during the preceding seven days, including both purchased and freely received meals [17]. However, this was not captured in the present freelists and therefore could not be examined as a potential contributor to the observed urban–rural differences.

Clustering analyses point in the same general direction. Higher food expenditure and higher education showed broader, more fragmented repertoires containing a wider range of market foods, while lower expenditure and rural settings were more tightly anchored around staple foods. Regional evidence supports this interaction between household access, preference, and policy-shaped food environments. In a six-country survey of 4116 Pacific adults, Buksh et al. found that greater household access and unhealthy-eating attitudes showed the strongest relationship with consumption of sugar-sweetened beverages and ultra-processed snacks [33]. Price showed smaller and less consistent associations, while household access was also closely related to fruit and vegetable consumption [33]. Thow et al.’s historical analyses of Fiji and Samoa provide a complementary structural perspective, showing that export-oriented and liberalising policies coincided with declining traditional root-crop availability and the increasing availability of refined cereals, while periods of agricultural investment and import substitution coincided with greater root-crop availability [34]. In Samoa, dietary shifts toward cereal remained after cyclone-related food aid and taro-blight recovery, even as root-crop production recovered, showing how changes in supply can become embedded in longer-term dietary patterns [34].

Together, the present associations and previous regional evidence support an interpretation in which consumption of imported food reflects the interaction of cultural preference with household resources and policy-shaped availability. Persistent local-food salience suggests that households may continue to value traditional foods even when the surrounding food environment favours imported alternatives, but whether constraints in the food environment limit the translation of these preferences into consumption requires direct investigation.

### 4.4. Cognitive Salience Is Not Dietary Intake

A key interpretive issue is the difference between what comes readily to mind and what is consumed in the greatest quantity. Existing Kiribati dietary data indicate near-universal consumption of bread and flour products, yet these items remained cognitively peripheral in the present freelists despite their widespread consumption [15,17]. Urban bread intake has been estimated at approximately 130 g per capita per day compared with approximately 13 g for chicken, despite chicken being much more prominent in our preference data. Similarly, sugar contributes substantially to dietary energy, with qualitative work documenting routine sugar use in drinks and foods, yet it was rarely listed spontaneously [6,17]. These discrepancies suggest that ingredients, condiments, and even certain staples may be behaviourally important while remaining cognitively backgrounded. A recent systematic review highlights this gap between stated preferences and objectively observed purchasing behaviour and argues for combining complementary measurement approaches [35]. Freelisting therefore adds a complementary dimension to dietary intake measures by identifying collective cultural prominence and shared preference. By identifying foods that are repeatedly and prominently recalled across participants, it reveals the cultural prominence and shared preference structures that underpin food choice, including foods that may remain highly valued despite being consumed less. Conversely, foods that are consumed frequently may be cognitively peripheral because they are habitual, ubiquitous, or treated as background ingredients. Therefore, used alongside dietary recall or purchasing data, freelisting can help distinguish what people eat from what they collectively value, providing a more complete account of the cultural and structural influences shaping food choice.

### 4.5. Priorities for Intervention Evaluation

Our findings instead suggest that intervention can benefit from building on existing cultural preference, as several locally produced foods still occupy a prominent place in Kiribati food culture. Table 3 shows that local food reaches rural and urban households by different routes. Rural respondents obtained it mainly by harvesting (88.3%) and fishing (87.9%), whereas urban respondents mainly bought it, at the fish market (65.5%) or elsewhere (82.0%). Interventions therefore cannot be uniform. In rural settings, the binding constraints are production, post-harvest loss and preservation, with 86.7% of rural respondents salting food and only 11.7% being able to refrigerate it. In urban settings the constraints sit in the retail supply chain, in price, and in the reliability of cold storage.

The objective now is to design interventions addressing these factors to determine whether they increase selection and consumption of culturally valued local foods. First, transport, cold-chain capacity, and coordinated supply could be evaluated as strategies to improve the reliable availability of fish and other perishable local foods between outer islands and South Tarawa. Second, local processing may offer ways to reduce preparation burden and seasonal losses. Breadfruit flour and other shelf-stable products have been proposed as ways to extend the usability of traditional crops, while babai and other local staples also offer opportunities for culturally grounded product development. Current practices remain mostly household-level, but scaling production to a local level could support dietary diversification and reduce consumption of imported goods [36,37,38,39]. Third, the low salience of added sugar, salt, and refined flour supports health communication and direct dietary assessments to establish which ingredients warrant further intervention. Fourth, 68% of respondents responsible for household food preparation were women, so women hold most of the practical knowledge about how local foods are prepared, stored and substituted, which is the knowledge these freelists captured. This argues for involving women as co-designers rather than only as recipients. In practice, this would mean women setting the criteria by which any new local product is judged to be acceptable, including taste, preparation time and fuel use; women recruited through existing community and church-based groups, which are the established route for reaching households in Kiribati; women represented in the governance of any local processing enterprise, so that income from it accrues to the households doing the work; and preparation burden measured as an outcome in its own right, since convenience is the constraint women report most directly. Finally, reforms of subsidies for traditional crops and local processing should be evaluated, as existing subsidies that encourage copra production are unsustainable [28].

### 4.6. Strengths, Limitations, and Future Research

This study has several strengths. First, the large sample size of 399 participants exceeds the recommended minimum of 20 for freelisting and cultural consensus analysis, enhancing the statistical robustness and reliability of the findings [21]. The inclusion of respondents from both urban and rural communities, despite considerable logistical challenges, allowed for meaningful comparison across settings and helped highlight the complexity of food preferences within Kiribati. Second, the combination of Smith’s S analysis, cultural consensus modelling, and hierarchical clustering provided a comprehensive view of how foods are valued and conceptualised. Few studies in the Pacific distinguish cognitive salience from dietary intake, making these findings a valuable contribution to the literature. The methodological design also prioritised culturally sensitive data collection, with local research assistants conducting interviews in respondents’ preferred languages.

Several limitations should be acknowledged. The study’s cross-sectional design captures perceptions at a single point in time. Second, data were collected prior to the COVID-19 pandemic, which disrupted Pacific food systems and may have changed access and preferences. Third, participants were recruited in South Tarawa, North Tarawa and Butaritari, and 85% of respondents were classified as urban. This limits the precision of rural subgroup comparisons, although this study showed a clearly discernible difference. Lack of coverage of other remote communities, other island groups and Banaba also limits the generalisability across Kiribati’s wider geographical settings. Due to constraints both in resources and timing (the country closed its borders during the COVID-19 pandemic, limiting future data collection for subsequent years) we were only able to obtain a limited number of outer island communities. Fourth, scree-plot inflexion points involve analyst judgement even when applied consistently. Fifth, freelisting measures spontaneous cognitive prominence rather than intake volume and does not capture meal source or preparation, preventing distinction between home-prepared foods and foods obtained outside the household. It may also underrepresent ingredients such as sugar, flour, and salt. Responses may additionally be affected by social desirability bias, while standardisation decisions may influence the resulting lists. Sixth, national household expenditure evidence indicates that eating away from home forms part of food provisioning in Kiribati. In the 2019/20 Household Income and Expenditure Survey, 64% of households reported lunch away from home during the preceding seven days; these meals included both purchased and freely received food. This was, unfortunately, almost impossible to capture using the scope of our research [17]. In terms of the effect on our study, the effect may be minimal as freelisting here captures preference, not actual consumption patterns. Seventh, the regression analyses were exploratory and use proportions derived from favourite food lists rather than observed purchasing. Finally, the study did not measure food prices, local availability, or the alternatives respondents faced, so explanations based on cost, supply, storage, or convenience are drawn from previous work and author observations rather than tested here.

Future work should link cultural salience with repeated dietary recall, household expenditure or purchase data, food prices, availability, and qualitative interviews on convenience and cultural meaning. Longitudinal or intervention studies could then test whether improving the affordability, shelf life, and convenience of locally valued foods changes actual purchasing and consumption. The COVID-19 period also provides a natural context for examining how urban and rural food systems respond to import shocks and whether culturally retained food knowledge increases resilience.

## 5. Conclusions

Food preferences in Kiribati reflect a nutrition transition in which continuity and change coexist. Fish, breadfruit, and coconut remain culturally salient across preference, tradition, and frequently eaten domains, while rice has become deeply integrated into everyday food repertoires, including rural concepts of traditional food. Urban food preferences are more standardised and strongly oriented toward market-supplied items, whereas rural repertoires retain greater traditional diversity. The association between high food-expenditure burden and preference for imported or processed food suggests that preference may be shaped by food-system constraints, although the mechanisms underlying these associations cannot be established from the present cross-sectional data.

This highlights an important distinction for nutrition-transition research: consumption data describe what households eat, whereas cultural salience can reveal what communities collectively value. The continued prominence of local staples indicates that culturally valued foods remain an important part of food preferences, providing a culturally grounded basis for the design of future food-choice interventions. Subsequent studies linking these preferences to prices, preparation and measured intake can assess which interventions improve dietary outcomes in the communities concerned.

## Figures and Tables

**Table 1:** Villages categorised into urban and rural localities based on the enumeration map provided by the Kiribati National Statistics Office.

Locality	Villages
Urban	Bikenibeu, Eita, Abarao, Betio, Ambo, Ananau, Taborio, Temwaiku, Bairiki, Teaoraereke, and Bonriki
Rural	Bikaati, Tabonuea, Buota, Nabeina, Marenanuka, and Buaraiki

**Table 2:** Study population characteristics, broken down into overall population, urban, and rural localities. Percentages are shown to 2 significant figures and may add up to more than 100% due to rounding. Values are n (%).

Characteristic	Overall	Rural	Urban
**Age in years (n = 399)**	**(n = 399)**	**(n = 60)**	**(n = 339)**
18–20	5 (1.3%)	0 (0%)	5 (1.5%)
21–30	80 (20%)	22 (37%)	58 (17%)
31–40	105 (26%)	20 (33%)	85 (25%)
41–50	110 (28%)	12 (20%)	98 (29%)
51–60	60 (15%)	5 (8.3%)	55 (16%)
61–70	32 (8%)	1 (1.7%)	31 (9.1%)
71–80	7 (1.8%)	0 (0%)	7 (2.1%)
**Education (n = 392)**	**(n = 392)**	**(n = 55)**	**(n = 337)**
None	14 (3.6%)	4 (7.3%)	10 (3%)
Primary	57 (15%)	6 (11%)	51 (15%)
Secondary	112 (29%)	17 (31%)	95 (28%)
High School	132 (34%)	19 (35%)	113 (34%)
Technical College	18 (4.6%)	3 (5.5%)	15 (4.5%)
University	59 (15%)	6 (11%)	53 (16%)
**Sex (n = 399)**	**(n = 399)**	**(n = 60)**	**(n = 339)**
Female	270 (68%)	35 (58%)	235 (69%)
Male	129 (32%)	25 (42%)	104 (31%)
**Food Expenditure (n = 394)**	**(n = 394)**	**(n = 60)**	**(n = 339)**
<25%	8 (2%)	2 (3.4%)	6 (1.8%)
25–50%	98 (25%)	28 (47%)	70 (21%)
51–75%	173 (44%)	29 (49%)	144 (43%)
>75%	115 (29%)	0 (0%)	115 (34%)
**Median household size (n = 399)**	**(n = 399)**	**(n = 60)**	**(n = 339)**
	7 (5, 9)	6 (5, 8)	7 (5, 9)

**Table 3:** Local food acquisition, sharing, preservation and cold storage practices, broken down into overall population, rural, and urban localities. Denominators differ between measures because not all respondents answered every question. Percentages are shown to one decimal place and may add up to more than 100% due to rounding.

Question and Measure	Overall	Rural	Urban
**Sources of local food**			
Q35 Local food harvesting	240/397 (60.5%)	53/60 (88.3%)	187/337 (55.5%)
Q23 Fish market	230/391 (58.8%)	12/58 (20.7%)	218/333 (65.5%)
Q23 Fishing or family fishing	192/391 (49.1%)	51/58 (87.9%)	141/333 (42.3%)
Q38 Purchased local food	303/394 (76.9%)	29/60 (48.3%)	274/334 (82.0%)
**Sharing of local food**			
Q36 Shared local food	188/395 (47.6%)	20/60 (33.3%)	168/335 (50.1%)
Q37 Received local food	154/395 (39.0%)	13/60 (21.7%)	141/335 (42.1%)
**Food preservation methods**			
Q39 Refrigeration preservation	221/396 (55.8%)	7/60 (11.7%)	214/336 (63.7%)
Q39 Salting preservation	169/396 (42.7%)	52/60 (86.7%)	117/336 (34.8%)
**Appliance ownership and use**			
Q54 Refrigerator ownership	177/396 (44.7%)	7/60 (11.7%)	170/336 (50.6%)
Q55 Refrigerator use	189/396 (47.7%)	7/60 (11.7%)	182/336 (54.2%)
Q56 Freezer ownership	205/397 (51.6%)	16/60 (26.7%)	189/337 (56.1%)
Q57 Freezer use	219/397 (55.2%)	17/60 (28.3%)	202/337 (59.9%)
**Food access constraints**			
Q51 Less-preferred food due to ease or cost	170/366 (46.4%)	13/59 (22.0%)	157/307 (51.1%)

**Table 4:** Consensus analysis of urban and rural subgroups showing eigenvalue ratios. FF = favourite foods, TF = traditional foods, T3 = top-three foods eaten most often. Eigenvalues are absolute and scale with the number of respondents, so their magnitude should not be compared across groups of different size. The first-to-second eigenvalue ratio is independent of group size and is the basis for the 3:1 criterion. Competence and RMSR are proportions, not percentages.

Domain	Group	First Eigenvalue	Second Eigenvalue	Eigenvalue Ratio	Mean Competence	Competence Range	RMS Residual
FF	All	18.611	2.311	8.053	0.931	0.831–0.988	0.023
FF	Urban	17.028	2.214	7.691	0.924	0.822–0.986	0.025
FF	Rural	6.827	1.207	5.655	0.880	0.747–0.974	0.044
TF	All	18.105	2.563	7.065	0.905	0.733–0.974	0.036
TF	Urban	16.560	2.444	6.776	0.898	0.716–0.973	0.038
TF	Rural	6.349	1.766	3.595	0.817	0.654–0.934	0.083
T3	All	19.177	2.811	6.821	0.960	0.810–0.993	0.026
T3	Urban	17.660	2.690	6.565	0.959	0.802–0.992	0.027
T3	Rural	6.901	1.877	3.677	0.886	0.521–0.977	0.070

## Data Availability

The data presented in this study are available on reasonable request from the corresponding author [J.P.C.], subject to the ethical and data-sharing restrictions governing the original household survey.

## Supplementary Materials

The following supporting information can be downloaded at: https://www.mdpi.com/article/10.3390/foods15193499/s1: Supplementary File S1, parent-study sample-size calculation and enumeration-area household sampling protocol; Supplementary File S2, household survey including freelisting items; Supplementary File S3, complete overall freelist salience rankings and scree plots; Supplementary File S4, complete urban/rural salience rankings and scree plots; Supplementary File S5, classification of freelisted foods.
