# Supplementary material for: Food Salience and the Nutrition Transition in Kiribati: Urban–Rural and Socioeconomic Patterning of Food Preferences
Source: Foods. 2026 Sep 29;15(19):3499. doi: 10.3390/foods15193499 (PMC13665049; doi:10.3390/foods15193499)
Supplement: Supplementary file 1 [file foods-15-03499-s001.zip › Supplementary File S3.pdf]

Supplementary File S3

3.1 FF Salience

Saliency and frequency of mention of all 133 food items collected in the Favourite Food Freelist, ordered by descending salience score. Italics indicate the foods that were included in the analysis

CODE	MeanSalience	SumSalience	SmithsS
Fish	0.23083588	81.48506494	0.2042232204
Rice	0.21488679	59.09386724	0.1481049304
Breadfruit	0.17422526	35.89040404	0.0899508873
Chicken	0.16032203	25.33088023	0.0634859154
Babai	0.17104892	14.36810967	0.0360102999
Coconut	0.14763169	14.32027417	0.0358904115
Egg	0.14313337	10.01933622	0.0251111183
Sausage	0.13734237	9.47662338	0.0237509358
Pork	0.13045100	7.69660895	0.0192897467
Pumpkin	0.12782588	7.66955267	0.0192219365
Cabbage	0.10420990	6.56522367	0.0164541946
Banana	0.12952036	5.95793651	0.0149321717
Cassava	0.10026761	5.51471861	0.0138213499
Corned Beef	0.16798115	5.37539683	0.0134721725
Potatoes	0.14028379	4.62936508	0.0116024187
Crab	0.11543831	4.61753247	0.0115727631
Bread	0.16225521	4.54314574	0.0113863302
Beef Meat	0.12357053	3.95425685	0.0099104182
Lamb	0.13028561	3.77828283	0.0094693805
Seashells	0.12171717	3.65151515	0.0091516670
Vegetables	0.13192363	3.43001443	0.0085965274
Tinned Fish	0.17443482	3.13982684	0.0078692402

CODE	MeanSalience	SumSalience	SmithsS
Raw Fish	0.15973266	3.03492063	0.0076063174
Toddy	0.10693442	2.88722944	0.0072361640
Octopus	0.17077441	2.56161616	0.0064200906
Noodles	0.10208033	2.44992785	0.0061401700
Ham-Pork	0.14950397	2.39206349	0.0059951466
Pandanus	0.14571158	2.33138528	0.0058430709
Pawpaw	0.10055524	2.31277056	0.0057964175
Prawn	0.14155393	2.26486291	0.0056763482
Sugar	0.08991342	2.24783550	0.0056336729
Tinned food	0.12424643	2.23643579	0.0056051022
Lobster	0.09197030	2.20728716	0.0055320480
Mutton	0.13453102	2.01796537	0.0050575573
Flour	0.13436267	2.01544012	0.0050512284
Bero Fig	0.11075036	1.99350649	0.0049962569
Doughnuts	0.11184534	1.90137085	0.0047653405
Taro	0.08986463	1.88715729	0.0047297175
Tomatoes	0.10112045	1.71904762	0.0043083900
Seaweed	0.11014911	1.65223665	0.0041409440
Coconut cream/milk	0.12197802	1.58571429	0.0039742213
Sliced Pork	0.19464286	1.55714286	0.0039026137
Cucumber	0.11312576	1.47063492	0.0036858018
Nouo	0.11268176	1.46486291	0.0036713356
Salad	0.10677656	1.38809524	0.0034789354
Eggplant	0.13726551	1.37265512	0.0034402384
Papaya	0.09908425	1.28809524	0.0032283089
Ibo worm	0.12285714	1.22857143	0.0030791264
Green leaves	0.09867725	1.18412698	0.0029677368
Clam	0.16768707	1.17380952	0.0029418785
Tinned Meat	0.14661797	1.17294372	0.0029397086

CODE	MeanSalience	SumSalience	SmithsS
Pizza	0.14255952	1.14047619	0.0028583363
Tapioca	0.11914683	0.95317460	0.0023889088
Boiled fish	0.29629630	0.88888889	0.0022277917
Smoked Fish	0.20932540	0.83730159	0.0020985002
Salted Fish	0.15619048	0.78095238	0.0019572741
Lamb Neck	0.10496805	0.73477633	0.0018415447
Bacon	0.14063492	0.70317460	0.0017623424
Noni	0.11616162	0.69696970	0.0017467912
Water Cress	0.06825397	0.61428571	0.0015395632
Biscuit	0.11699856	0.58499278	0.0014661473
Fried Fish	0.19393939	0.58181818	0.0014581909
Tea	0.07113095	0.56904762	0.0014261845
Coffee	0.05811287	0.52301587	0.0013108167
Chilli	0.09828283	0.49141414	0.0012316144
Ice-Cream	0.11904762	0.47619048	0.0011934598
Lettuce	0.09523810	0.47619048	0.0011934598
Seashell (Koumara)	0.23611111	0.47222222	0.0011835143
Clam - te were	0.15079365	0.45238095	0.0011337868
Taro leaves	0.11111111	0.44444444	0.0011138959
Popup	0.05986395	0.41904762	0.0010502447
Sweet Potato (koumara)	0.08238095	0.41190476	0.0010323428
Breads	0.06190476	0.37142857	0.0009308987
Lentils (dhal)	0.12380952	0.37142857	0.0009308987
Duck	0.18492063	0.36984127	0.0009269205
Melon	0.12222222	0.36666667	0.0009189641
Pie	0.12063492	0.36190476	0.0009070295
Pancake	0.07216450	0.36082251	0.0009043171
Onion	0.16666667	0.33333333	0.0008354219

CODE	MeanSalience	SumSalience	SmithsS
Meat	0.15595238	0.31190476	0.0007817162
Cheese	0.06150072	0.30750361	0.0007706857
Buka leaves	0.13888889	0.27777778	0.0006961849
Watermelon	0.08994709	0.26984127	0.0006762939
Katiobuki (flour	0.12943723	0.25887446	0.0006488082
Carrot	0.08316498	0.24949495	0.0006253006
Tiuee	0.12121212	0.24242424	0.0006075796
Mantis shrimp	0.11785714	0.23571429	0.0005907626
coconut and tuee)	0.11558442	0.23116883	0.0005793705
Chaya	0.22222222	0.22222222	0.0005569479
Chips	0.11060606	0.22121212	0.0005544163
Milk	0.05506854	0.22027417	0.0005520656
Eel	0.09325397	0.18650794	0.0004674384
Pudding	0.08928571	0.17857143	0.0004475474
Milk (Sunquick)	0.05846561	0.17539683	0.0004395910
Shellfish(koikoi)	0.16666667	0.16666667	0.0004177109
Confectionary (Cakes)	0.16666667	0.16666667	0.0004177109
Coconut shoots (young)	0.16666667	0.16666667	0.0004177109
Coconut syrup	0.08116883	0.16233766	0.0004068613
Beans	0.07777778	0.15555556	0.0003898635
Ham-Pork	0.05185185	0.15555556	0.0003898635
Oat porridge	0.14285714	0.14285714	0.0003580380
Ice-cream	0.13888889	0.13888889	0.0003480925
Lime/Lemon	0.13888889	0.13888889	0.0003480925
Butter	0.04540645	0.13621934	0.0003414018
Fruits (local)	0.06746032	0.13492063	0.0003381470
Noni leaves	0.06547619	0.13095238	0.0003282015
Seafood	0.06515152	0.13030303	0.0003265740

CODE	MeanSalience	SumSalience	SmithsS
Milo	0.06190476	0.12380952	0.0003102996
Goose	0.06071429	0.12142857	0.0003043323
Grilled Fish	0.11111111	0.11111111	0.0002784740
Blue peas	0.05555556	0.11111111	0.0002784740
Coconut water	0.11111111	0.11111111	0.0002784740
Baked beans	0.10714286	0.10714286	0.0002685285
Scone	0.09523810	0.09523810	0.0002386920
Curried meat	0.04761905	0.09523810	0.0002386920
Milkfish	0.09523810	0.09523810	0.0002386920
Chocolate	0.09523810	0.09523810	0.0002386920
Cordial	0.04523810	0.09047619	0.0002267574
Fried Chicken	0.08333333	0.08333333	0.0002088555
Ginger	0.08333333	0.08333333	0.0002088555
Spaghetti	0.07142857	0.07142857	0.0001790190
Mango	0.07142857	0.07142857	0.0001790190
Crisps (snack)	0.07142857	0.07142857	0.0001790190
Roti	0.06666667	0.06666667	0.0001670844
Kabuki (things made with flour)	0.06666667	0.06666667	0.0001670844
Drumstick	0.05555556	0.05555556	0.0001392370
Pineapple	0.05555556	0.05555556	0.0001392370
Lamb neck	0.04761905	0.04761905	0.0001193460
Ice-block	0.04761905	0.04761905	0.0001193460
Meat pie	0.03571429	0.03571429	0.0000895095
Koikoi nanti	0.02777778	0.02777778	0.0000696185
Fried Rice	0.01904762	0.01904762	0.0000477384

3.2 TF Salience

Saliency and frequency of mention of all 133 food items collected in the Traditional Food Freelist, ordered by descending salience score. Italics indicate the foods that were included in the analysis

CODE	MeanSalience	SumSalience	SmithsS
Breadfruit	0.21258708	69.30338828	0.1736927025
Fish	0.21156422	64.52708680	0.1617220221
Coconut	0.17776944	41.59804917	0.1042557623
Babai	0.18165602	38.14776335	0.0956084294
Pumpkin	0.13869214	25.24196914	0.0632630806
Pandanus	0.14129724	19.92291042	0.0499321063
Banana	0.13086849	16.88203463	0.0423108637
Pawpaw	0.14915270	11.18645244	0.0280362216
Papaya	0.13913396	9.32197525	0.0233633465
Toddy	0.11226591	7.74634810	0.0194144063
Cabbage	0.13903404	7.22977023	0.0181197249
Crab	0.13682010	6.70418470	0.0168024679
Bero fig	0.14268005	6.27792208	0.0157341405
Seashells	0.11053055	5.74758852	0.0144049838
Cassava	0.11808862	5.66825397	0.0142061503
Octopus	0.10521236	3.89285714	0.0097565342
Rice	0.24724868	3.70873016	0.0092950631
Sweet Potato (koumara)	0.14668110	3.22698413	0.0080876795
Noni	0.12858364	3.21459096	0.0080566190
Eggplant	0.11357490	3.06652237	0.0076855197
Lobster	0.10503118	2.94087302	0.0073706091
Water Cress	0.11686508	2.80476190	0.0070294785

CODE	MeanSalience	SumSalience	SmithsS
Pig	0.12626606	2.65158730	0.0066455822
Potatoes	0.14724556	2.50317460	0.0062736206
Vegetables (local)	0.11492063	2.29841270	0.0057604328
Taro Leaves	0.09860381	2.26788767	0.0056839290
Tapioca	0.12268908	2.08571429	0.0052273541
Chicken	0.10219697	2.04393939	0.0051226551
Seagrass	0.13148148	1.97222222	0.0049429128
Ibo worm	0.11228869	1.90890776	0.0047842300
Local green leaves	0.07447926	1.71302309	0.0042932909
Nouo	0.09993536	1.69890110	0.0042578975
Drumstick leaves	0.11185185	1.67777778	0.0042049568
Taro	0.21309524	1.27857143	0.0032044397
Prawn	0.11908730	1.19087302	0.0029846442
Tiuee	0.11691518	1.05223665	0.0026371846
Buka leaves	0.10978836	0.98809524	0.0024764292
Bele	0.13594104	0.95158730	0.0023849306
Coconut syrup	0.09418248	0.94182484	0.0023604633
Coconut cream	0.10370370	0.93333333	0.0023391813
Cucumber	0.09303351	0.83730159	0.0020985002
Eel	0.09986772	0.59920635	0.0015017703
Egg	0.11190476	0.55952381	0.0014023153
Dried Pandanus	0.50000000	0.50000000	0.0012531328
Melon	0.15079365	0.45238095	0.0011337868
Tomatoes	0.11111111	0.44444444	0.0011138959
Koikoi	0.14761905	0.44285714	0.0011099177
Spinach	0.10257937	0.41031746	0.0010283646
Flour	0.20000000	0.40000000	0.0010025063
Clam - te were	0.09226190	0.36904762	0.0009249314
Pancake	0.09166667	0.36666667	0.0009189641

CODE	MeanSalience	SumSalience	SmithsS
Dried Breadfruit	0.33333333	0.33333333	0.0008354219
Salted fish	0.33333333	0.33333333	0.0008354219
Seafood	0.07175325	0.28701299	0.0007193308
Breads	0.28571429	0.28571429	0.0007160759
Smoked fish	0.26666667	0.26666667	0.0006683375
Sausage	0.12500000	0.25000000	0.0006265664
Blue peas	0.11904762	0.23809524	0.0005967299
Doughnut	0.23809524	0.23809524	0.0005967299
Kanawa leaves	0.11111111	0.22222222	0.0005569479
Pudding	0.22222222	0.22222222	0.0005569479
Tinned fish	0.20000000	0.20000000	0.0005012531
Fermented toddy	0.17857143	0.17857143	0.0004475474
Passion fruit	0.17777778	0.17777778	0.0004455583
Carrot	0.16666667	0.16666667	0.0004177109
Watermelon	0.16666667	0.16666667	0.0004177109
Crab Coconut	0.14285714	0.14285714	0.0003580380
Lime/Lemon	0.13888889	0.13888889	0.0003480925
Koumwara seashell	0.10714286	0.10714286	0.0002685285
Taro	0.05119048	0.10238095	0.0002565939
Coconut water	0.04761905	0.09523810	0.0002386920
Noni leaves	0.08333333	0.08333333	0.0002088555
Chilli	0.03896104	0.07792208	0.0001952934
Mango	0.03769841	0.07539683	0.0001889645
Clam	0.07142857	0.07142857	0.0001790190
Pumpkin tips	0.06666667	0.06666667	0.0001670844
Vegetables	0.06060606	0.06060606	0.0001518949
Turtle	0.05714286	0.05714286	0.0001432152
Noni juice	0.04761905	0.04761905	0.0001193460
Koikoi nanti	0.03571429	0.03571429	0.0000895095

CODE	MeanSalience	SumSalience	SmithsS
Coconut fudge	0.03571429	0.03571429	0.0000895095
Sweet Potato leaves	0.02857143	0.02857143	0.0000716076
Manta Rays	0.02857143	0.02857143	0.0000716076
Coconut sprout	0.02777778	0.02777778	0.0000696185
Shark	0.01904762	0.01904762	0.0000477384

3.3 T3 Salience

Saliency and frequency of mention of all 54 food items collected in the Top Three Food question, ordered by descending salience score. Italics indicate the foods that were included in the analysis.

CODE	MeanSalience	SumSalience	SmithsS
Rice	0.3978938	144.8333333	0.362990810
Fish	0.3680556	141.3333333	0.354218881
Breadfruit	0.2767296	29.3333333	0.073517126
Chicken	0.2477064	27.0000000	0.067669173
Coconut	0.2232143	12.5000000	0.031328321
Babai	0.3235294	5.5000000	0.013784461
Pumpkin	0.2777778	3.3333333	0.008354219
Vegetables	0.2000000	3.0000000	0.007518797
Doughnuts	0.2962963	2.6666667	0.006683375
Tinned Fish	0.2222222	2.0000000	0.005012531
Beef Meat	0.2619048	1.8333333	0.004594820
Cabbage	0.1875000	1.5000000	0.003759398
Sugar	0.1875000	1.5000000	0.003759398
Raw Fish	0.3333333	1.3333333	0.003341688
Sausage	0.3333333	1.3333333	0.003341688
Pork	0.2916667	1.1666667	0.002923977
Octopus	0.2916667	1.1666667	0.002923977

CODE	MeanSalience	SumSalience	SmithsS
Noodles	0.2916667	1.1666667	0.002923977
Banana	0.3333333	1.0000000	0.002506266
Bread	0.2500000	1.0000000	0.002506266
Tinned food	0.2000000	1.0000000	0.002506266
Lentils (dhal)	0.5000000	1.0000000	0.002506266
Potatoes	0.2083333	0.8333333	0.002088555
Green leaves	0.2083333	0.8333333	0.002088555
Toddy	0.2222222	0.6666667	0.001670844
Cassava	0.1666667	0.6666667	0.001670844
Seashells	0.3333333	0.6666667	0.001670844
Flour	0.3333333	0.6666667	0.001670844
Shellfish(koikoi)	0.5000000	0.5000000	0.001253133
Bacon	0.5000000	0.5000000	0.001253133
Salted Fish	0.2500000	0.5000000	0.001253133
Boiled fish	0.5000000	0.5000000	0.001253133
Meat	0.2500000	0.5000000	0.001253133
Tinned Meat	0.1666667	0.5000000	0.001253133
Taro	0.5000000	0.5000000	0.001253133
Mutton	0.2500000	0.5000000	0.001253133
Tea	0.5000000	0.5000000	0.001253133
Oat porridge	0.5000000	0.5000000	0.001253133
Corned Beef	0.1666667	0.3333333	0.000835422
Taro leaves	0.1666667	0.3333333	0.000835422
Breads	0.3333333	0.3333333	0.000835422
Goose	0.1666667	0.1666667	0.000417711
Fried Chicken	0.1666667	0.1666667	0.000417711
Coffee	0.1666667	0.1666667	0.000417711
Water Cress	0.1666667	0.1666667	0.000417711
Crab	0.1666667	0.1666667	0.000417711

CODE	MeanSalience	SumSalience	SmithsS
Noni	0.1666667	0.1666667	0.000417711
Lamb	0.1666667	0.1666667	0.000417711
Ice-block	0.1666667	0.1666667	0.000417711
Sliced Pork	0.1666667	0.1666667	0.000417711
Pawpaw	0.1666667	0.1666667	0.000417711
Pancake	0.1666667	0.1666667	0.000417711
Clam	0.1666667	0.1666667	0.000417711
Seaweed	0.1666667	0.1666667	0.000417711
