# Supplementary material for: Food Salience and the Nutrition Transition in Kiribati: Urban–Rural and Socioeconomic Patterning of Food Preferences
Source: Foods. 2026 Sep 29;15(19):3499. doi: 10.3390/foods15193499 (PMC13665049; doi:10.3390/foods15193499)
Supplement: Supplementary file 1 [file foods-15-03499-s001.zip › Supplementary File S1.pdf]

Supplementary File S1

1.1 Power calculations

Power calculations were carried out using the online Statulator tool 1 for power estimates. The sample calculation was carried out using the households as the units for the population size, using the 2015 census2 as reference.

Betio: n =17,330 people | 2,293 households

South Tarawa: n =39,058 people | 5,584 households

North Tarawa: n = 6,629 people | 1,128 households

Butaritari: n = 3,224 people | 624 households

Total households = 2,293 (Betio) + 5,584 (S Tarawa) + 1,128 (N Tarawa) + 624 (Butaritari) = 9,629 households taken as 'total population' size.

Sample calculation: n= 9,629, 95% confidence interval, expected proportion of households with food insecurity at 0.50*, expected response rate at 90% = sample size of 411 households.

Notes:

*this assumption was selected because the study is exploratory, and therefore the assumed actual number of households with food insecurity is unknown.

1.2 EA sampling technique

Survey Instruction Manual (Selecting Households)

When selecting households, please look first at the Enumeration Area map provided. I'll be using ST13 in Eita as an example below.

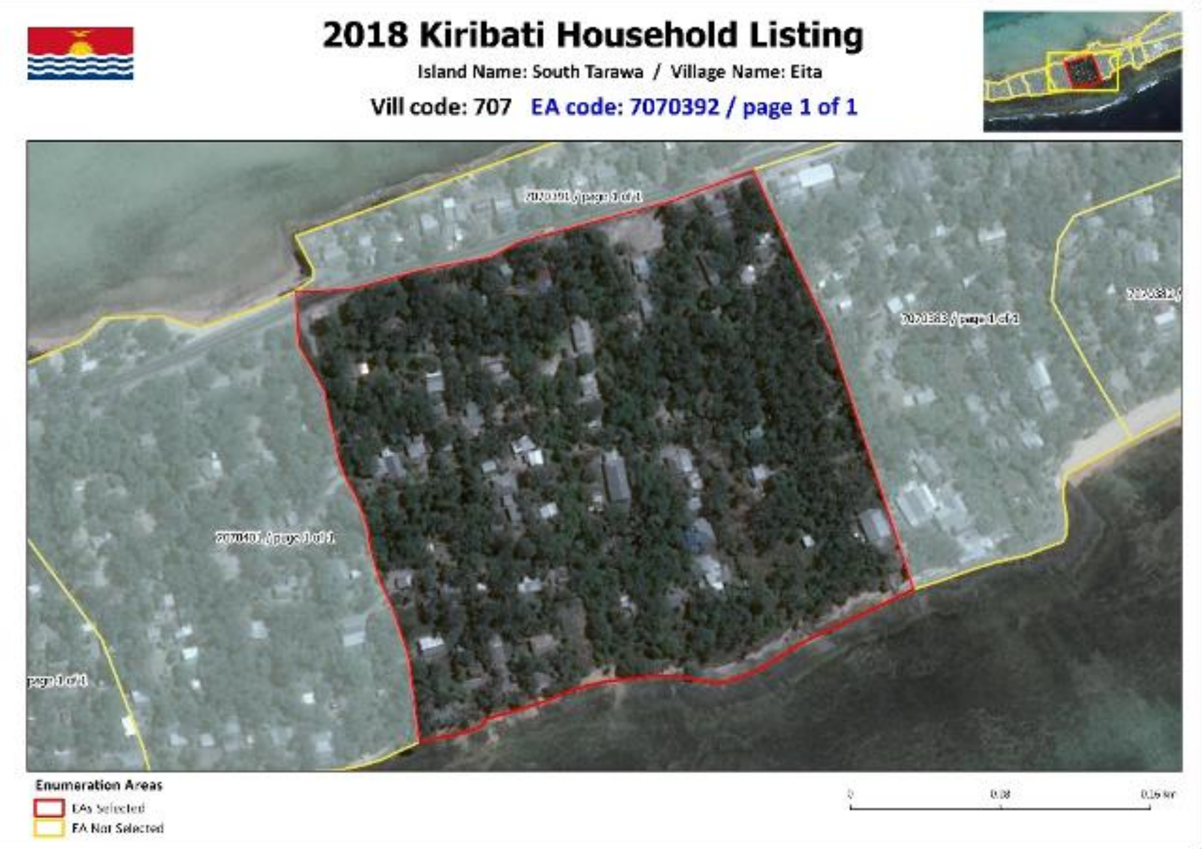

As you can observe, this is roughly a square-shaped area, on the ocean side of South Tarawa. It holds 50 households and has a population of 281.

We will now discuss **the selection process,** summarised in a picture below.

1. Choose a 'corner' of the area provided
2. Start at the first household you find at that corner
3. Once a survey is completed choose a direction following the shape of the area.
4. SKIP TWO HOUSEHOLDS – this is crucial, as it will introduce randomness in the sample.
5. Do the next household, and go back to step 4.
6. Once the next edge of an area is reached, do an 's-bend', and turn back up.

There are exceptions to the rule:

Exception 1: if a household selected turns out to be an office/store/shop/government building/maneaba/church or refuses to participate then please move to the next household. After that, go back to step 4.

Exception 2: If the person who is responsible for taking care of food is NOT at the house, then you should go back to that household at a later time or date.

Exception 3: If there are aggressive dogs, etc, kindly do not put yourself in danger and move to the next households.

Exception 4: If the house has two floors or more, please go to the top floor of one house, and the bottom floor of the next house.

Map Showing a typical Enumeration Area pattern of choosing

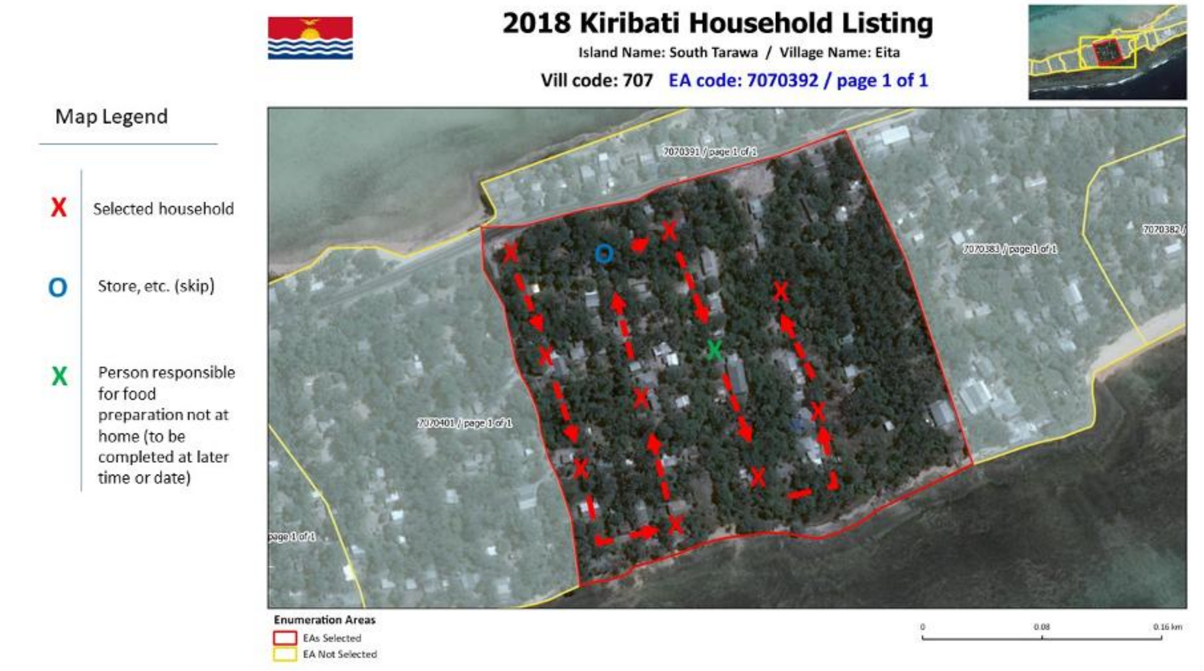

If you follow these basic principles when doing an Enumeration area, it should be quite straightforward.

Please remember:

Do NOT go outside the EA when doing surveys

Do NOT skip selected households unless forced to

WRITE your names on the Surveys (not on the consent form)

Fill in the ENTIRE survey. Don’t leave things empty unless required.

If Unsure of how to answer, write an answer. Better to have more information than less.
