# Supplementary material for: Food Salience and the Nutrition Transition in Kiribati: Urban–Rural and Socioeconomic Patterning of Food Preferences
Source: Foods. 2026 Sep 29;15(19):3499. doi: 10.3390/foods15193499 (PMC13665049; doi:10.3390/foods15193499)
Supplement: Supplementary file 1 [file foods-15-03499-s001.zip › Supplementary File S2.pdf]

KIRIBATI HOUSEHOLD SURVEY

QUT School of Public Health and Social Work

Date ____________________________ **Enumeration Area Code** _________________

Dear interviewer, remember to ask the questions as accurately as possible in English or i-Kiribati so that their meaning isn't changed. Additionally, remember to try to talk to the most person most involved in food preparation in the household.

1. Are you the person in the house who is responsible for food preparation?

**YES**	**NO**

2. If not, who is responsible for it? Could we speak to that person today?

**YES**	**NO**

Background Information

In this section we will ask questions about you.

3. Sex (***DO NOT ASK***)

**MALE**	**FEMALE**

4. Can you tell me your age?

18-20	21-30	31-40
41-50	51-60	61-70
71- 80	81+	

5. Can you tell me your marital status?

Married	Single
Living together as a couple	
Widowed	Separated or Divorced

6. What is your Religious affiliation?

➔ *If **Other** please specify* ____________________

Roman Catholic	Kiribati Uniting Church	Protestant
Seventh Day Adventist	Mormon	Bahá'í Faith
Assemblies of God	Jehovah's Witnesses	**Other**

7. What is the highest level of education you obtained?

None	Primary
Secondary	High School
Technical College	University

8. Total number of people in your household?

Answer __________________________

9. On which island were you born?

Answer __________________________

10. How long have you lived on **this** island?
(The island where you are carrying out the survey)

< 5 years	5 - 10 years
10 – 20 years	20 years +

11. What brought you to **this** island?

➔ *If **Other** specify here* ____________________________

Work	School
Health	Family
**Other**	

12. Do you plan on staying long-term on this island?

➔ *If **NO**, answer, why do you plan to move?*

____

YES	**NO**

13. What do you do for a living?

➔ *If **Full time worker, Part time worker or 'Other'** please ask for place of employment (eg. Government office)*

____

**Full time worker**	**Part time worker**
Fisherman	Make and sell crafts
Student	Retired
Unemployed	**Other**

14. What does your household spend most of its money on?
➔ *If **Other** please ask and specify below:*

____

Food	Education
Travel	Health
**Other**	

15. How much of your income does your family spend on food?

> 75%	51% – 75%
25% - 50%	< 25%

16. Does **anyone** in your household… (select any)

➔ *If **Food Garden** is selected, ask what is grown*
____

➔ *If **Keep chickens** is selected, ask how many* ___________

➔ *If **Keep Pigs** is selected, ask how many* ___________

➔ *If **Go fishing** is selected, ask how often, and where (lagoon, open sea).*
____

➔ *If **Other** please specify what kind of subsistence activity is practiced*
____

**Go fishing**	**Keeps a food garden***	**Keep pigs***
Keep a *Babai* Pit	**Harvests food from trees *** (eg. Breadfruit)	**Keep *chickens**
**Other**		

17. How many children under 18 years old live in your household?

Insert number ________________________

18. How many **full-time** workers in paid employment live in your household?

Insert number ________________________

19. How many **part-time** workers in paid employment live in your household?

Insert number ________________________

Characterising the Food System

This section asks questions about the food you eat in your household. How much is local versus store food, how you get that food, and whether you share food.

Freelisting 1: Favourite Foods

20. What are your favourite foods? Please tell me which ones they are.

← *List the foods mentioned.* ***DO NOT PROMPT OR GIVE SUGGESTIONS.*** *Give 1 minute (not more) for replies. Time yourself. List them* ***IN ORDER*** *of mention.*

21. What three foods do you eat most often?

1. ______________________________
2. ______________________________
3. ______________________________

22. How many times **per week** do you eat fresh fish?

< 1 time or rarely	1 or 2 times
3 or 4 times	5 or 6 times
Every day	Don't know

23. Where do you get your fish?

*Answer*______________________________

24. Are there somedays when you can't get fish?

➔ *If **YES** ask why* ______________________________

**YES**	NO

25. What do you do when you can't get fish?

*Answer*______________________________

26. How many times per week do you eat instant noodles?

< 1 time or rarely	1 or 2 times
3 or 4 times	5 or 6 times
Every day	Don't know

27. How many times per week do you eat chicken

< 1 time or rarely	1 or 2 times
3 or 4 times	5 or 6 times
Every day	Don't know

28. How many times per week do you eat **tinned** fish?

< 1 time or rarely	1 or 2 times
3 or 4 times	5 or 6 times
Every day	Don't know

29. How many times per week do you eat **tinned** meat?

< 1 time or rarely	1 or 2 times
3 or 4 times	5 or 6 times
Every day	Don't know

30. Think about all the food you ate in the past **month.** How much of it was **local** food?

None	Less than Half
About half	More than Half
All of it	Don't know

31. What about all the food you ate in the past **year.** How much of that food was **local** food?

None	Less than Half
About half	More than Half
All of it	Don't know

32. Do you think you eat more local food compared to last year, or less?

➔ *If **more** or **less**, why?* ________________________
Eg. Local food is more expensive, etc.

**Less**	About the Same
**More**	Don't know

33. If you went to a feast (eg. Wedding, etc), what sort of food would you bring with you?

Local	Imported
Mixed	Don't know

Freelisting 2: Traditional Foods

34. What are your Traditional foods? Please tell me which ones they are.

← *List the foods mentioned.* ***DO NOT PROMPT OR GIVE SUGGESTIONS.*** *Give 1 minute (not more) for replies. Time yourself. List them* ***IN ORDER*** *of mention.*

Food Sources and Food Sharing

35. Do you or any members of your household harvest local food?

**YES**	NO

➔ If **Yes**, what do you harvest? ______________________

36. In the last year, did you or your household **share local food** with people outside your household?

➔ Why? ___________________________________

➔ With whom? ____________________________

Always	Often
Sometimes	Rarely
Never	We used to but not any more

37. In the last year, did you or your household **receive local food** from people outside your household?

➔ Why? ___________________________________

➔ From who? ____________________________

Always	Often
Sometimes	Rarely
Never	We used to but not any more

38. In the last year, did you or your household pay for local food?

Always	Often
Sometimes	Rarely
Never	We used to but not any more

39. In the last year, did you ever preserve any local food? *(circle all appropriate answers)*

➔ *If **Other**, please write method*

____

Boiling for a long time	Salting
Refrigeration	**Other**

40. In the last year, did you or your household ever **share store food** with people outside your household?

➔ *If Always, Often, Sometimes or Rarely*

Why? _________________________________

With whom? ____________________________

**Always**	**Often**
**Sometimes**	**Rarely**
Never	We used to but not any more

41. In the last year, did you or your household **receive store food** from outside your household?

➔ *If Always, Often, Sometimes or Rarely*

Why? _________________________________

From whom? ____________________________

**Always**	**Often**
**Sometimes**	**Rarely**
Never	We used to but not any more

Local and store Food availability/access

This section asks questions about the availability of local and store food in your locality, and whether your household has access to it.

42. In the last year, were you able to get **local** food most of the time?

YES	**NO**

➔ If **No**, why? ______________________________________

➔ If **No**, how did you cope? ___________________________

43. How would you say your ability to get local food was last year compared to previous years?

Easier	Harder
Same	
Much easier	Much harder

44. In the last year, were you able to get **store** food most of the time?

YES	**NO**

➔ If **No**, why? ______________________________________

➔ If **No**, how did you cope? ___________________________

45. How would you say your ability to get store food was last year compared to previous years?

Easier	Harder
Same	
Much easier	Much harder

Food security & insecurity

This section asks questions about being able to get enough food, and getting healthy food for your household.

46. In the **last month**, did you ever worry that the food for you and your family might run out before you could get more?

Often	Sometimes
Never	Don't know

47. What about in the **last year?**

Often	Sometimes
Never	Don't know

48. In the **last month,** did the food you bought or received go bad or expire and you couldn't buy or get more?

Often	Sometimes
Never	Don't know

49. What about in the **last year?**

Often	Sometimes
Never	Don't know

50. In the **last month**, did you or anyone in your household **skip meals, reduce portions, or feel hungry** because you did not have enough food?

Often	Sometimes
Never	Don't know

51. In the **last month**, did you or anyone in your household **eat foods that you do not like as much**, because they are easier or cheaper to get?

Often	Sometimes
Never	Don't know

52. In the **last month**, did you or anyone in your household eat **lower quality foods** because you couldn't get higher quality, healthier foods?

Often	Sometimes
Never	Don't know

53. In the last year, do you feel that your diet was healthy or unhealthy for you?

Healthy	Unhealthy
Somewhere in between	

54. Do you own a **refrigerator**?

➔ *If **No** go to 51.*

YES	**NO**

55. Do use a refrigerator to store food? (eg. Neighbours)

YES	NO

56. Do you own a **freezer**?

➔ *If **No** go to 52.*

YES	**NO**

57. Do use a freezer to store food? (eg. Neighbours)

YES	NO

Health and Illness

This section asks questions about health problems in your household. ***We assure you that all information gathered will be confidential and that nobody will be able to trace this information to you****.*

58. Have you or anyone in your household experienced or been told you have **high blood sugar or diabetes?** — YES | NO

59. Have you or has anyone in your household experienced or been told you have **high blood pressure?** — YES | NO

60. Have you or has anyone in your household experienced or been told you have **low iron/ anaemia?** — YES | NO

61. Have you or has anyone in your household experienced or been told you have **eye problems?** — YES | NO

62. In the last **year**, have you or anyone in your household had **fish poisoning (Ciguatera)?** — YES | NO

63. In the last **month**, have you or anyone in your household been sick with **diarrhoea?** — YES | NO

64. In the **last month,** have or anyone in your household you had to go to the bathroom with **diarrhoea** more than three times in a day? — YES | NO

65. Does someone in your household suffer from **infectious/communicable diseases?** — YES | NO

66. Does someone in your household suffer from **skin diseases?** — YES | NO

67. Have you or has anyone in your household experienced or been told you have any other illness? — **YES** | NO

➔ If **Yes**, what type of sickness ________________________

68. In your opinion, what should be done to help people in your island to get enough of the food they want to eat?

➔ *Write a short answer*

*Answer*______________________

Water and Sanitation

This section asks questions about your sources of water, and your methods of sanitation.

69. Where do you get **most** of your drinking water?

➔ *If **Other*** ___________________________________

PUB	Pipe system
Well	Water Pump
Rainwater	**Other**

70. Do you treat your drinking water?

➔ If **Yes**, how do you treat?
____

**YES**	NO

71. What type of toilet do you use in your household?

➔ *If **Other*** ___________________________________

PUB flush toilet	Other flush toilet
Water latrine	*Kamkamka*
Beach	Bush
Sea	**Other**

Essential Items

*In the **past year**, how often, if ever, has your household had to go **without**t the following things?*

† if person uses solar power as main source of electricity, please write down
**if possible specify order of medical preference (eg. Traditional, then clinic)*

72. Go without **clean water**?

➔ *Always (10 times out of 10), Often (7-9 times), Sometimes (4-6 times), Rarely (1-3 times), Never (0 times out of 10)*

Always	Often	Sometimes
Rarely	Never	Don't Know

73. Go without **electricity**? †

➔ *Always (10 times out of 10), Often (7-9 times), Sometimes (4-6 times), Rarely (1-3 times), Never (0 times out of 10)*

Always	Often	Sometimes
Rarely	Never	Don't Know

74. Go without **medicines or medical treatment**?*

➔ *Always (10 times out of 10), Often (7-9 times), Sometimes (4-6 times), Rarely (1-3 times), Never (0 times out of 10)*

Always	Often	Sometimes
Rarely	Never	Don't Know

75. Go without **enough income** to buy what you need?

➔ *Always (10 times out of 10), Often (7-9 times), Sometimes (4-6 times), Rarely (1-3 times), Never (0 times out of 10)*

Always	Often	Sometimes
Rarely	Never	Don't Know

76. Go without **fuel** (Copra/ Kerosene) too cook with?

➔ *Always (10 times out of 10), Often (7-9 times), Sometimes (4-6 times), Rarely (1-3 times), Never (0 times out of 10)*

Always	Often	Sometimes
Rarely	Never	Don't Know

Environmental Concerns

77. Do you worry about climate change?

Always	Often	Sometimes
Rarely	Never	Don't Know

78. Does your community experience coastal erosion?

YES	NO

79. Do you experience rainwater flooding in your community?

YES	NO

80. Do you experience drought in your community?

YES	NO

81. Do you experience seawater intrusion in your community?

YES	NO

82. Are there any other environmental concerns in your community that worry you?

Answer ________________________

CONCLUSION

This brings us to the end of the survey. Thank you for your time and for your participation. Should you wish to contact us again, kindly refer to the contact details on the information sheet.
