# Supplementary material for: Food Salience and the Nutrition Transition in Kiribati: Urban–Rural and Socioeconomic Patterning of Food Preferences
Source: Foods. 2026 Sep 29;15(19):3499. doi: 10.3390/foods15193499 (PMC13665049; doi:10.3390/foods15193499)
Supplement: Supplementary file 1 [file foods-15-03499-s001.zip › Supplementary File S5.pdf]

Supplementary File S5. Classification of freelisted food items

Each item in the favourite-foods freelist was assigned a category, a source, and a level of processing. These tags define the three regression outcomes: the proportion of a respondent's listed items that were imported (source = imported), processed (processing = processed), and traditional (source = local and processing = unprocessed).

The three categories are not mutually exclusive and do not sum to one. Rice counts towards both the imported and processed proportions, while potatoes, being imported but unprocessed, count towards the imported proportion only. Chicken could not be assigned to a single source or processing level, as both locally raised and imported chicken are consumed, and so counts towards none of the three proportions. Items that respondents listed but that do not appear in this table were likewise counted towards none of the three categories while being retained in the denominator. Each proportion therefore represents a share of the respondent's entire list rather than of classified items alone.

Food item	Category	Source	Processing*	Notes
**Fish**	Protein	Local	Unprocessed	Daily traditional staple
**Rice**	Carbohydrate	Imported	Processed	Main imported staple
**Breadfruit**	Carbohydrate	Local	Unprocessed	Traditional starch
**Chicken**	Protein	Local or imported	Variable	Both locally raised and imported chicken are consumed; not assigned to a single category
**Coconut**	Fat/Plant	Local	Unprocessed	Versatile traditional food
**Babai**	Carbohydrate	Local	Unprocessed	Traditional swamp taro, ceremonial
**Pumpkin**	Vegetable	Local	Unprocessed	Often home-grown
**Cabbage**	Vegetable	Local	Unprocessed	Often home-grown
**Pork**	Protein	Local	Unprocessed	Ceremonial meat
**Sausage**	Protein	Imported	Processed	Modern store meat
**Egg**	Protein	Local	Unprocessed	Often home-grown
**Banana**	Fruit	Local	Unprocessed	Local fruit
**Cassava**	Carbohydrate	Local	Unprocessed	Root crop
**Crab**	Protein	Local	Unprocessed	Occasional seafood
**Corned Beef**	Protein	Imported	Processed	Highly processed
**Potatoes**	Carbohydrate	Imported	Unprocessed	Purchased starch
**Bread**	Carbohydrate	Imported	Processed	Western staple
**Seashells**	Protein	Local	Unprocessed	Foraged or seasonal
**Pawpaw**	Fruit	Local	Unprocessed	Seasonal local fruit
**Toddy**	Beverage	Local	Unprocessed	Cultural beverage
**Lamb**	Protein	Imported	Processed	Imported red meat
**Beef Meat**	Protein	Imported	Processed	Usually frozen or canned
**Flour**	Carbohydrate	Imported	Processed	Used in baking
**Biscuit**	Snack	Imported	Processed	Packaged sweet/savoury snack
**Tea**	Beverage	Imported	Processed	Caffeine, non-nutritive
**Milo**	Beverage	Imported	Processed	Sweet powdered drink
**Soft Drink**	Beverage	Imported	Processed	Sugary soda drink
**Sugar**	Sweetener	Imported	Processed	Added to drinks and food
**Noodles**	Carbohydrate	Imported	Processed	Instant food, low nutrition
**Butter**	Fat/Dairy	Imported	Processed	Imported dairy spread

** "Unprocessed" includes minimally processed items, for example those ground by hand within the household.*
