# Supplementary material for: Food Salience and the Nutrition Transition in Kiribati: Urban–Rural and Socioeconomic Patterning of Food Preferences
Source: Foods. 2026 Sep 29;15(19):3499. doi: 10.3390/foods15193499 (PMC13665049; doi:10.3390/foods15193499)
Supplement: Supplementary file 1 [file foods-15-03499-s001.zip › Supplementary File S4.pdf]

Supplementary File S4

4.1 FF urban

Saliency and frequency of mention of all 128 food items collected in the urban category, ordered by descending salience score. Italics indicate the foods that were included in the analysis.

GROUPING	CODE	MeanSalience	SumSalience	SmithsS
Urban	*Fish*	0.23362509	69.38665224	0.2046803901
Urban	*Rice*	0.21043999	46.71767677	0.1378102560
Urban	*Breadfruit*	0.16518604	26.92532468	0.0794257365
Urban	*Chicken*	0.16152686	23.09834055	0.0681366978
Urban	*Coconut*	0.15051430	11.74011544	0.0346316090
Urban	*Egg*	0.14430863	9.81298701	0.0289468643
Urban	*Sausage*	0.13734237	9.47662338	0.0279546412
Urban	*Babai*	0.14962722	8.97763348	0.0264826946
Urban	Pork	0.13264042	7.02994228	0.0207372929
Urban	Cabbage	0.09845624	5.71046176	0.0168450199
Urban	Pumpkin	0.11762658	5.41082251	0.0159611284
Urban	Cassava	0.09859708	5.32424242	0.0157057299
Urban	Corned Beef	0.17233169	4.99761905	0.0147422391
Urban	Potatoes	0.14028379	4.62936508	0.0136559442
Urban	Banana	0.13024943	4.55873016	0.0134475816
Urban	Bread	0.15935176	4.14314574	0.0122216689
Urban	Beef Meat	0.12357053	3.95425685	0.0116644745
Urban	Lamb	0.13028561	3.77828283	0.0111453771
Urban	Crab	0.10917579	3.71197691	0.0109497844
Urban	Vegetables	0.13666727	3.28001443	0.0096755588
Urban	Seashells	0.11510513	3.22294372	0.0095072086

GROUPING	CODE	MeanSalience	SumSalience	SmithsS
Urban	Raw Fish	0.15973266	3.03492063	0.0089525682
Urban	Tinned Fish	0.16508785	2.80649351	0.0082787419
Urban	Toddy	0.10437807	2.60945166	0.0076974975
Urban	Octopus	0.17503608	2.45050505	0.0072286285
Urban	Ham-Pork	0.15312169	2.29682540	0.0067752962
Urban	Prawn	0.14155393	2.26486291	0.0066810115
Urban	Sugar	0.09250241	2.22005772	0.0065488428
Urban	Lobster	0.09307046	2.14062049	0.0063145147
Urban	Mutton	0.13453102	2.01796537	0.0059527002
Urban	Noodles	0.10350497	1.96659452	0.0058011638
Urban	Tinned food	0.11655604	1.74834055	0.0051573468
Urban	Tomatoes	0.10112045	1.71904762	0.0050709369
Urban	Taro	0.08483405	1.69668110	0.0050049590
Urban	Pandanus	0.14117364	1.69408369	0.0049972970
Urban	Flour	0.15335170	1.68686869	0.0049760138
Urban	Seaweed	0.11014911	1.65223665	0.0048738544
Urban	Coconut cream/milk	0.12197802	1.58571429	0.0046776233
Urban	Sliced Pork	0.19464286	1.55714286	0.0045933418
Urban	Doughnuts	0.11001855	1.54025974	0.0045435391
Urban	Bero Fig	0.09882636	1.48239538	0.0043728477
Urban	Pawpaw	0.09256403	1.48102453	0.0043688039
Urban	Nouo	0.11651635	1.39819625	0.0041244727
Urban	Salad	0.10677656	1.38809524	0.0040946762
Urban	Eggplant	0.13726551	1.37265512	0.0040491302
Urban	Green leaves	0.09867725	1.18412698	0.0034930000
Urban	Tinned Meat	0.15803958	1.10627706	0.0032633541
Urban	Cucumber	0.10373016	1.03730159	0.0030598867
Urban	Ibo worm	0.11005291	0.99047619	0.0029217587
Urban	Pizza	0.13117914	0.91825397	0.0027087138

GROUPING	CODE	MeanSalience	SumSalience	SmithsS
Urban	Boiled fish	0.29629630	0.88888889	0.0026220911
Urban	Clam	0.14801587	0.88809524	0.0026197500
Urban	Smoked Fish	0.20932540	0.83730159	0.0024699162
Urban	Lamb Neck	0.10496805	0.73477633	0.0021674818
Urban	Salted Fish	0.18333333	0.73333333	0.0021632252
Urban	Bacon	0.14063492	0.70317460	0.0020742614
Urban	Noni	0.11616162	0.69696970	0.0020559578
Urban	Tapioca	0.13333333	0.66666667	0.0019665683
Urban	Papaya	0.08900227	0.62301587	0.0018378049
Urban	Water Cress	0.06825397	0.61428571	0.0018120523
Urban	Biscuit	0.11699856	0.58499278	0.0017256424
Urban	Fried Fish	0.19393939	0.58181818	0.0017162778
Urban	Chilli	0.09828283	0.49141414	0.0014495992
Urban	Tea	0.06938776	0.48571429	0.0014327855
Urban	Lettuce	0.09523810	0.47619048	0.0014046917
Urban	Seashell (Koumara)	0.23611111	0.47222222	0.0013929859
Urban	Coffee	0.05843254	0.46746032	0.0013789390
Urban	Clam - te were	0.15079365	0.45238095	0.0013344571
Urban	Taro leaves	0.11111111	0.44444444	0.0013110456
Urban	Ice-Cream	0.14285714	0.42857143	0.0012642225
Urban	Popup	0.05986395	0.41904762	0.0012361287
Urban	Sweet Potato (koumara)	0.08238095	0.41190476	0.0012150583
Urban	Duck	0.18492063	0.36984127	0.0010909772
Urban	Melon	0.12222222	0.36666667	0.0010816126
Urban	Pie	0.12063492	0.36190476	0.0010675657
Urban	Pancake	0.07216450	0.36082251	0.0010643732
Urban	Onion	0.16666667	0.33333333	0.0009832842
Urban	Cheese	0.06150072	0.30750361	0.0009070903

GROUPING	CODE	MeanSalience	SumSalience	SmithsS
Urban	Breads	0.05761905	0.28809524	0.0008498385
Urban	Buka leaves	0.13888889	0.27777778	0.0008194035
Urban	Watermelon	0.08994709	0.26984127	0.0007959919
Urban	Katiobuki (flour	0.12943723	0.25887446	0.0007636415
Urban	Carrot	0.08316498	0.24949495	0.0007359733
Urban	Tiuee	0.12121212	0.24242424	0.0007151158
Urban	Mantis shrimp	0.11785714	0.23571429	0.0006953224
Urban	coconut and tuee)	0.11558442	0.23116883	0.0006819140
Urban	Chips	0.11060606	0.22121212	0.0006525431
Urban	Milk	0.05506854	0.22027417	0.0006497763
Urban	Eel	0.09325397	0.18650794	0.0005501709
Urban	Meat	0.17857143	0.17857143	0.0005267594
Urban	Pudding	0.08928571	0.17857143	0.0005267594
Urban	Milk (Sunquick)	0.05846561	0.17539683	0.0005173948
Urban	Shellfish(koikoi)	0.16666667	0.16666667	0.0004916421
Urban	Confectionary (Cakes)	0.16666667	0.16666667	0.0004916421
Urban	Coconut syrup	0.08116883	0.16233766	0.0004788722
Urban	Ham-Pork	0.05185185	0.15555556	0.0004588659
Urban	Oat porridge	0.14285714	0.14285714	0.0004214075
Urban	Ice-cream	0.13888889	0.13888889	0.0004097017
Urban	Lime/Lemon	0.13888889	0.13888889	0.0004097017
Urban	Butter	0.04540645	0.13621934	0.0004018270
Urban	Fruits (local)	0.06746032	0.13492063	0.0003979960
Urban	Noni leaves	0.06547619	0.13095238	0.0003862902
Urban	Seafood	0.06515152	0.13030303	0.0003843747
Urban	Milo	0.06190476	0.12380952	0.0003652198
Urban	Goose	0.06071429	0.12142857	0.0003581964
Urban	Grilled Fish	0.11111111	0.11111111	0.0003277614
Urban	Blue peas	0.05555556	0.11111111	0.0003277614

GROUPING	CODE	MeanSalience	SumSalience	SmithsS
Urban	Coconut water	0.11111111	0.11111111	0.0003277614
Urban	Baked beans	0.10714286	0.10714286	0.0003160556
Urban	Scone	0.09523810	0.09523810	0.0002809383
Urban	Curried meat	0.04761905	0.09523810	0.0002809383
Urban	Milkfish	0.09523810	0.09523810	0.0002809383
Urban	Cordial	0.04523810	0.09047619	0.0002668914
Urban	Beans	0.08888889	0.08888889	0.0002622091
Urban	Fried Chicken	0.08333333	0.08333333	0.0002458210
Urban	Ginger	0.08333333	0.08333333	0.0002458210
Urban	Spaghetti	0.07142857	0.07142857	0.0002107038
Urban	Mango	0.07142857	0.07142857	0.0002107038
Urban	Roti	0.06666667	0.06666667	0.0001966568
Urban	Drumstick	0.05555556	0.05555556	0.0001638807
Urban	Pineapple	0.05555556	0.05555556	0.0001638807
Urban	Lamb neck	0.04761905	0.04761905	0.0001404692
Urban	Ice-block	0.04761905	0.04761905	0.0001404692
Urban	Meat pie	0.03571429	0.03571429	0.0001053519
Urban	Koikoi nanti	0.02777778	0.02777778	0.0000819403
Urban	Fried Rice	0.01904762	0.01904762	0.0000561877
Urban	Chocolate		0.00000000	0.0000000000
Urban	Chaya		0.00000000	0.0000000000
Urban	Coconut shoots (young)		0.00000000	0.0000000000
Urban	Lentils (dhal)		0.00000000	0.0000000000
Urban	Kabuki (things made with flour)		0.00000000	0.0000000000
Urban	Crisps (snack)		0.00000000	0.0000000000

4.2 FF rural

Saliency and frequency of mention of all 52 food items collected in the rural category, ordered by descending salience score. Italics indicate the foods that were included in the analysis.

GROUPING	CODE	MeanSalience	SumSalience	SmithsS
Rural	*Rice*	0.23351303	12.37619048	0.2062698413
Rural	*Fish*	0.21604308	12.09841270	0.2016402116
Rural	*Breadfruit*	0.20849022	8.96507937	0.1494179894
Rural	*Babai*	0.22460317	5.39047619	0.0898412698
Rural	*Coconut*	0.13579783	2.58015873	0.0430026455
Rural	*Pumpkin*	0.16133787	2.25873016	0.0376455026
Rural	*Chicken*	0.14883598	2.23253968	0.0372089947
Rural	Banana	0.12720058	1.39920635	0.0233201058
Rural	Crab	0.15092593	0.90555556	0.0150925926
Rural	Cabbage	0.17095238	0.85476190	0.0142460317
Rural	Pawpaw	0.11882086	0.83174603	0.0138624339
Rural	Pork	0.11111111	0.66666667	0.0111111111
Rural	Papaya	0.11084656	0.66507937	0.0110846561
Rural	Pandanus	0.15932540	0.63730159	0.0106216931
Rural	Bero Fig	0.17037037	0.51111111	0.0085185185
Rural	Tinned food	0.16269841	0.48809524	0.0081349206
Rural	Noodles	0.09666667	0.48333333	0.0080555556
Rural	Cucumber	0.14444444	0.43333333	0.0072222222
Rural	Seashells	0.21428571	0.42857143	0.0071428571
Rural	Bread	0.20000000	0.40000000	0.0066666667
Rural	Corned Beef	0.12592593	0.37777778	0.0062962963
Rural	Lentils (dhal)	0.12380952	0.37142857	0.0061904762
Rural	Doughnuts	0.12037037	0.36111111	0.0060185185

GROUPING	CODE	MeanSalience	SumSalience	SmithsS
Rural	Tinned Fish	0.33333333	0.33333333	0.0055555556
Rural	Flour	0.08214286	0.32857143	0.0054761905
Rural	Tapioca	0.09550265	0.28650794	0.0047751323
Rural	Clam	0.28571429	0.28571429	0.0047619048
Rural	Toddy	0.13888889	0.27777778	0.0046296296
Rural	Ibo worm	0.23809524	0.23809524	0.0039682540
Rural	Pizza	0.22222222	0.22222222	0.0037037037
Rural	Chaya	0.22222222	0.22222222	0.0037037037
Rural	Egg	0.10317460	0.20634921	0.0034391534
Rural	Cassava	0.19047619	0.19047619	0.0031746032
Rural	Taro	0.19047619	0.19047619	0.0031746032
Rural	Coconut shoots (young)	0.16666667	0.16666667	0.0027777778
Rural	Vegetables	0.07500000	0.15000000	0.0025000000
Rural	Meat	0.13333333	0.13333333	0.0022222222
Rural	Octopus	0.11111111	0.11111111	0.0018518519
Rural	Ham-Pork	0.09523810	0.09523810	0.0015873016
Rural	Chocolate	0.09523810	0.09523810	0.0015873016
Rural	Tea	0.08333333	0.08333333	0.0013888889
Rural	Breads	0.08333333	0.08333333	0.0013888889
Rural	Crisps (snack)	0.07142857	0.07142857	0.0011904762
Rural	Beans	0.06666667	0.06666667	0.0011111111
Rural	Tinned Meat	0.06666667	0.06666667	0.0011111111
Rural	Lobster	0.06666667	0.06666667	0.0011111111
Rural	Nouo	0.06666667	0.06666667	0.0011111111
Rural	Kabuki (things made with flour)	0.06666667	0.06666667	0.0011111111
Rural	Coffee	0.05555556	0.05555556	0.0009259259
Rural	Salted Fish	0.04761905	0.04761905	0.0007936508
Rural	Ice-Cream	0.04761905	0.04761905	0.0007936508

GROUPING	CODE	MeanSalience	SumSalience	SmithsS
Rural	Sugar	0.02777778	0.02777778	0.0004629630
Rural	Sausage		0.00000000	0.0000000000
Rural	Lamb neck		0.00000000	0.0000000000
Rural	Water Cress		0.00000000	0.0000000000
Rural	Spaghetti		0.00000000	0.0000000000
Rural	Pancake		0.00000000	0.0000000000
Rural	Prawn		0.00000000	0.0000000000
Rural	Beef Meat		0.00000000	0.0000000000
Rural	Fried Rice		0.00000000	0.0000000000
Rural	Lamb		0.00000000	0.0000000000
Rural	Mutton		0.00000000	0.0000000000
Rural	Eggplant		0.00000000	0.0000000000
Rural	Duck		0.00000000	0.0000000000
Rural	Potatoes		0.00000000	0.0000000000
Rural	Carrot		0.00000000	0.0000000000
Rural	Green leaves		0.00000000	0.0000000000
Rural	Noni		0.00000000	0.0000000000
Rural	Shellfish(koikoi)		0.00000000	0.0000000000
Rural	Goose		0.00000000	0.0000000000
Rural	Noni leaves		0.00000000	0.0000000000
Rural	Mantis shrimp		0.00000000	0.0000000000
Rural	Popup		0.00000000	0.0000000000
Rural	Bacon		0.00000000	0.0000000000
Rural	Watermelon		0.00000000	0.0000000000
Rural	Seaweed		0.00000000	0.0000000000
Rural	Fried Fish		0.00000000	0.0000000000
Rural	Smoked Fish		0.00000000	0.0000000000

GROUPING	CODE	MeanSalience	SumSalience	SmithsS
Rural	Raw Fish		0.00000000	0.0000000000
Rural	Grilled Fish		0.00000000	0.0000000000
Rural	Fried Chicken		0.00000000	0.0000000000
Rural	Tomatoes		0.00000000	0.0000000000
Rural	Boiled fish		0.00000000	0.0000000000
Rural	Chips		0.00000000	0.0000000000
Rural	Ice-cream		0.00000000	0.0000000000
Rural	Sweet Potato (koumara)		0.00000000	0.0000000000
Rural	Fruits (local)		0.00000000	0.0000000000
Rural	Onion		0.00000000	0.0000000000
Rural	Pudding		0.00000000	0.0000000000
Rural	Scone		0.00000000	0.0000000000
Rural	Milo		0.00000000	0.0000000000
Rural	Milk (Sunquick)		0.00000000	0.0000000000
Rural	Curried meat		0.00000000	0.0000000000
Rural	Cheese		0.00000000	0.0000000000
Rural	Chilli		0.00000000	0.0000000000
Rural	Coconut cream/milk		0.00000000	0.0000000000
Rural	Biscuit		0.00000000	0.0000000000
Rural	Sliced Pork		0.00000000	0.0000000000
Rural	Milkfish		0.00000000	0.0000000000
Rural	Lettuce		0.00000000	0.0000000000
Rural	Salad		0.00000000	0.0000000000
Rural	Meat pie		0.00000000	0.0000000000
Rural	Pie		0.00000000	0.0000000000
Rural	Eel		0.00000000	0.0000000000

GROUPING	CODE	MeanSalience	SumSalience	SmithsS
Rural	Lamb Neck		0.00000000	0.0000000000
Rural	Koikoi nanti		0.00000000	0.0000000000
Rural	Clam - te were		0.00000000	0.0000000000
Rural	Blue peas		0.00000000	0.0000000000
Rural	Confectionary (Cakes)		0.00000000	0.0000000000
Rural	Mango		0.00000000	0.0000000000
Rural	Drumstick		0.00000000	0.0000000000
Rural	Katiobuki (flour		0.00000000	0.0000000000
Rural	coconut and tuee)		0.00000000	0.0000000000
Rural	Taro leaves		0.00000000	0.0000000000
Rural	Buka leaves		0.00000000	0.0000000000
Rural	Seashell (Koumara)		0.00000000	0.0000000000
Rural	Ginger		0.00000000	0.0000000000
Rural	Pineapple		0.00000000	0.0000000000
Rural	Milk		0.00000000	0.0000000000
Rural	Butter		0.00000000	0.0000000000
Rural	Cordial		0.00000000	0.0000000000
Rural	Coconut water		0.00000000	0.0000000000
Rural	Ham-Pork		0.00000000	0.0000000000
Rural	Roti		0.00000000	0.0000000000
Rural	Baked beans		0.00000000	0.0000000000
Rural	Melon		0.00000000	0.0000000000
Rural	Ice-block		0.00000000	0.0000000000
Rural	Lime/Lemon		0.00000000	0.0000000000
Rural	Seafood		0.00000000	0.0000000000
Rural	Tiuee		0.00000000	0.0000000000

GROUPING	CODE	MeanSalience	SumSalience	SmithsS
Rural	Coconut syrup		0.00000000	0.0000000000
Rural	Oat porridge		0.00000000	0.0000000000

4.3 TF urban

Saliency and frequency of mention of all 80 food items collected in the urban category, ordered by descending salience score. Italics indicate the foods that were included in the analysis.

GROUPING	CODE	MeanSalience	SumSalience	SmithsS
Urban	*Breadfruit*	0.20945686	58.01954989	0.1711491147
Urban	*Fish*	0.21121007	57.66034799	0.1700895221
Urban	*Coconut*	0.18001056	37.08217616	0.1093869503
Urban	*Babai*	0.18036966	30.66284271	0.0904508635
Urban	*Pumpkin*	0.13135428	19.96585082	0.0588963151
Urban	*Pandanus*	0.14320482	17.75739816	0.0523817055
Urban	*Banana*	0.12494037	12.24415584	0.0361184538
Urban	Pawpaw	0.14349835	8.60990121	0.0253979387
Urban	Toddy	0.11151047	7.58271173	0.0223678812
Urban	Papaya	0.12783408	6.64737207	0.0196087672
Urban	Cabbage	0.14366300	6.46483516	0.0190703102
Urban	Crab	0.13411416	6.03513709	0.0178027643
Urban	Seashells	0.11387172	5.46584249	0.0161234292
Urban	Cassava	0.11964286	5.26428571	0.0155288664
Urban	Octopus	0.10521236	3.89285714	0.0114833544
Urban	Bero fig	0.13776655	3.71969697	0.0109725574
Urban	Noni	0.12858364	3.21459096	0.0094825692
Urban	Eggplant	0.11357490	3.06652237	0.0090457887
Urban	Water Cress	0.11686508	2.80476190	0.0082736339

GROUPING	CODE	MeanSalience	SumSalience	SmithsS
Urban	Lobster	0.10350794	2.58769841	0.0076333287
Urban	Potatoes	0.14724556	2.50317460	0.0073839959
Urban	Pig	0.12067460	2.41349206	0.0071194456
Urban	Rice	0.23039683	2.30396825	0.0067963665
Urban	Taro Leaves	0.09659230	2.12503053	0.0062685266
Urban	Vegetables (local)	0.11754850	2.11587302	0.0062415133
Urban	Seagrass	0.13148148	1.97222222	0.0058177647
Urban	Chicken	0.09873737	1.77727273	0.0052426924
Urban	Nouo	0.09993536	1.69890110	0.0050115077
Urban	Local green leaves	0.07570018	1.66540404	0.0049126963
Urban	Tapioca	0.12747253	1.65714286	0.0048883270
Urban	Drumstick leaves	0.11507937	1.61111111	0.0047525402
Urban	Ibo worm	0.10424464	1.56366966	0.0046125949
Urban	Sweet Potato (koumara)	0.13722222	1.37222222	0.0040478532
Urban	Taro	0.21309524	1.27857143	0.0037715971
Urban	Prawn	0.12305996	1.10753968	0.0032670787
Urban	Buka leaves	0.10978836	0.98809524	0.0029147352
Urban	Coconut cream	0.10370370	0.93333333	0.0027531957
Urban	Bele	0.16333333	0.81666667	0.0024090462
Urban	Coconut syrup	0.08848559	0.79637030	0.0023491749
Urban	Cucumber	0.09920635	0.69444444	0.0020485087
Urban	Tiuee	0.11203463	0.56017316	0.0016524282
Urban	Egg	0.11190476	0.55952381	0.0016505127
Urban	Dried Pandanus	0.50000000	0.50000000	0.0014749263
Urban	Melon	0.15079365	0.45238095	0.0013344571

GROUPING	CODE	MeanSalience	SumSalience	SmithsS
Urban	Koikoi	0.14761905	0.44285714	0.0013063633
Urban	Eel	0.08650794	0.43253968	0.0012759283
Urban	Tomatoes	0.13888889	0.41666667	0.0012291052
Urban	Spinach	0.10257937	0.41031746	0.0012103760
Urban	Clam - te were	0.09226190	0.36904762	0.0010886360
Urban	Pancake	0.09166667	0.36666667	0.0010816126
Urban	Dried Breadfruit	0.33333333	0.33333333	0.0009832842
Urban	Salted fish	0.33333333	0.33333333	0.0009832842
Urban	Seafood	0.07175325	0.28701299	0.0008466460
Urban	Smoked fish	0.26666667	0.26666667	0.0007866273
Urban	Sausage	0.12500000	0.25000000	0.0007374631
Urban	Blue peas	0.11904762	0.23809524	0.0007023458
Urban	Kanawa leaves	0.11111111	0.22222222	0.0006555228
Urban	Pudding	0.22222222	0.22222222	0.0006555228
Urban	Tinned fish	0.20000000	0.20000000	0.0005899705
Urban	Flour	0.20000000	0.20000000	0.0005899705
Urban	Passion fruit	0.17777778	0.17777778	0.0005244182
Urban	Carrot	0.16666667	0.16666667	0.0004916421
Urban	Watermelon	0.16666667	0.16666667	0.0004916421
Urban	Lime/Lemon	0.13888889	0.13888889	0.0004097017
Urban	Koumwara seashell	0.10714286	0.10714286	0.0003160556
Urban	Coconut water	0.04761905	0.09523810	0.0002809383
Urban	Noni leaves	0.08333333	0.08333333	0.0002458210
Urban	Chilli	0.03896104	0.07792208	0.0002298586
Urban	Mango	0.03769841	0.07539683	0.0002224095
Urban	Pumpkin tips	0.06666667	0.06666667	0.0001966568

GROUPING	CODE	MeanSalience	SumSalience	SmithsS
Urban	Taro	0.06666667	0.06666667	0.0001966568
Urban	Vegetables	0.06060606	0.06060606	0.0001787789
Urban	Turtle	0.05714286	0.05714286	0.0001685630
Urban	Noni juice	0.04761905	0.04761905	0.0001404692
Urban	Koikoi nanti	0.03571429	0.03571429	0.0001053519
Urban	Coconut fudge	0.03571429	0.03571429	0.0001053519
Urban	Sweet Potato leaves	0.02857143	0.02857143	0.0000842815
Urban	Manta Rays	0.02857143	0.02857143	0.0000842815
Urban	Coconut sprout	0.02777778	0.02777778	0.0000819403
Urban	Shark	0.01904762	0.01904762	0.0000561877
Urban	Clam		0.00000000	0.0000000000
Urban	Crab Coconut		0.00000000	0.0000000000
Urban	Breads		0.00000000	0.0000000000
Urban	Doughnut		0.00000000	0.0000000000
Urban	Fermented toddy		0.00000000	0.0000000000

4.4 TF rural

Saliency and frequency of mention of all 40 food items collected in the rural category, ordered by descending salience score. Italics indicate the foods that were included in the analysis.

GROUPING	CODE	MeanSalience	SumSalience	SmithsS
Rural	*Breadfruit*	0.23028242	11.28383838	0.1880639731
Rural	*Babai*	0.18712302	7.48492063	0.1247486772
Rural	*Fish*	0.21458559	6.86673882	0.1144456469

GROUPING	CODE	MeanSalience	SumSalience	SmithsS
Rural	*Pumpkin*	0.17587061	5.27611833	0.0879353054
Rural	*Banana*	0.14960899	4.63787879	0.0772979798
Rural	*Coconut*	0.16128118	4.51587302	0.0752645503
Rural	*Papaya*	0.17830688	2.67460317	0.0445767196
Rural	*Pawpaw*	0.17177008	2.57655123	0.0429425204
Rural	*Bero fig*	0.15048383	2.55822511	0.0426370851
Rural	*Pandanus*	0.12738307	2.16551227	0.0360918711
Rural	*Sweet Potato (koumara)*	0.15456349	1.85476190	0.0309126984
Rural	Rice	0.28095238	1.40476190	0.0234126984
Rural	Cabbage	0.10927644	0.76493506	0.0127489177
Rural	Crab	0.16726190	0.66904762	0.0111507937
Rural	Tiuee	0.12301587	0.49206349	0.0082010582
Rural	Tapioca	0.10714286	0.42857143	0.0071428571
Rural	Cassava	0.10099206	0.40396825	0.0067328042
Rural	Lobster	0.11772487	0.35317460	0.0058862434
Rural	Ibo worm	0.17261905	0.34523810	0.0057539683
Rural	Breads	0.28571429	0.28571429	0.0047619048
Rural	Seashells	0.07043651	0.28174603	0.0046957672
Rural	Chicken	0.13333333	0.26666667	0.0044444444
Rural	Pig	0.23809524	0.23809524	0.0039682540
Rural	Doughnut	0.23809524	0.23809524	0.0039682540
Rural	Flour	0.20000000	0.20000000	0.0033333333
Rural	Vegetables (local)	0.09126984	0.18253968	0.0030423280
Rural	Fermented toddy	0.17857143	0.17857143	0.0029761905
Rural	Eel	0.16666667	0.16666667	0.0027777778
Rural	Toddy	0.16363636	0.16363636	0.0027272727
Rural	Coconut syrup	0.14545455	0.14545455	0.0024242424

GROUPING	CODE	MeanSalience	SumSalience	SmithsS
Rural	Cucumber	0.07142857	0.14285714	0.0023809524
Rural	Taro Leaves	0.14285714	0.14285714	0.0023809524
Rural	Crab Coconut	0.14285714	0.14285714	0.0023809524
Rural	Bele	0.06746032	0.13492063	0.0022486772
Rural	Prawn	0.08333333	0.08333333	0.0013888889
Rural	Clam	0.07142857	0.07142857	0.0011904762
Rural	Drumstick leaves	0.06666667	0.06666667	0.0011111111
Rural	Local green leaves	0.04761905	0.04761905	0.0007936508
Rural	Taro	0.03571429	0.03571429	0.0005952381
Rural	Tomatoes	0.02777778	0.02777778	0.0004629630
Rural	Potatoes		0.00000000	0.0000000000
Rural	Water Cress		0.00000000	0.0000000000
Rural	Seagrass		0.00000000	0.0000000000
Rural	Egg		0.00000000	0.0000000000
Rural	Eggplant		0.00000000	0.0000000000
Rural	Koikoi		0.00000000	0.0000000000
Rural	Spinach		0.00000000	0.0000000000
Rural	Pumpkin tips		0.00000000	0.0000000000
Rural	Nouo		0.00000000	0.0000000000
Rural	Pancake		0.00000000	0.0000000000
Rural	Noni		0.00000000	0.0000000000
Rural	Sweet Potato leaves		0.00000000	0.0000000000
Rural	Blue peas		0.00000000	0.0000000000
Rural	Tinned fish		0.00000000	0.0000000000
Rural	Turtle		0.00000000	0.0000000000
Rural	Octopus		0.00000000	0.0000000000

GROUPING	CODE	MeanSalience	SumSalience	SmithsS
Rural	Manta Rays		0.00000000	0.0000000000
Rural	Shark		0.00000000	0.0000000000
Rural	Dried Pandanus		0.00000000	0.0000000000
Rural	Dried Breadfruit		0.00000000	0.0000000000
Rural	Noni juice		0.00000000	0.0000000000
Rural	Coconut water		0.00000000	0.0000000000
Rural	Melon		0.00000000	0.0000000000
Rural	Kanawa leaves		0.00000000	0.0000000000
Rural	Taro		0.00000000	0.0000000000
Rural	Buka leaves		0.00000000	0.0000000000
Rural	Sausage		0.00000000	0.0000000000
Rural	Coconut cream		0.00000000	0.0000000000
Rural	Koikoi nanti		0.00000000	0.0000000000
Rural	Coconut sprout		0.00000000	0.0000000000
Rural	Clam - te were		0.00000000	0.0000000000
Rural	Passion fruit		0.00000000	0.0000000000
Rural	Lime/Lemon		0.00000000	0.0000000000
Rural	Carrot		0.00000000	0.0000000000
Rural	Mango		0.00000000	0.0000000000
Rural	Chilli		0.00000000	0.0000000000
Rural	Koumwara seashell		0.00000000	0.0000000000
Rural	Noni leaves		0.00000000	0.0000000000
Rural	Pudding		0.00000000	0.0000000000

GROUPING	CODE	MeanSalience	SumSalience	SmithsS
Rural	Watermelon		0.00000000	0.0000000000
Rural	Salted fish		0.00000000	0.0000000000
Rural	Smoked fish		0.00000000	0.0000000000
Rural	Seafood		0.00000000	0.0000000000
Rural	Coconut fudge		0.00000000	0.0000000000
Rural	Vegetables		0.00000000	0.0000000000

4.5 T3 urban

Saliency and frequency of mention of all 52 food items collected in the urban category, ordered by descending salience score. Italics indicate the foods that were included in the analysis.

GROUPING	CODE	MeanSalience	SumSalience	SmithsS
Urban	*Fish*	0.3785569	124.1666667	0.366273353
Urban	*Rice*	0.3988095	122.8333333	0.362340216
Urban	*Chicken*	0.2484568	26.8333333	0.079154376
Urban	*Breadfruit*	0.2633745	21.3333333	0.062930187
Urban	*Coconut*	0.2200000	11.0000000	0.032448378
Urban	Vegetables	0.2051282	2.6666667	0.007866273
Urban	Pumpkin	0.2857143	2.0000000	0.005899705
Urban	Beef Meat	0.2619048	1.8333333	0.005408063
Urban	Tinned Fish	0.2083333	1.6666667	0.004916421
Urban	Doughnuts	0.2777778	1.6666667	0.004916421
Urban	Cabbage	0.1875000	1.5000000	0.004424779
Urban	Babai	0.2500000	1.5000000	0.004424779
Urban	Sugar	0.1875000	1.5000000	0.004424779
Urban	Raw Fish	0.3333333	1.3333333	0.003933137

GROUPING	CODE	MeanSalience	SumSalience	SmithsS
Urban	Sausage	0.3333333	1.3333333	0.003933137
Urban	Pork	0.2916667	1.1666667	0.003441495
Urban	Octopus	0.2916667	1.1666667	0.003441495
Urban	Potatoes	0.2083333	0.8333333	0.002458210
Urban	Green leaves	0.2083333	0.8333333	0.002458210
Urban	Toddy	0.2222222	0.6666667	0.001966568
Urban	Cassava	0.1666667	0.6666667	0.001966568
Urban	Banana	0.2500000	0.5000000	0.001474926
Urban	Shellfish(koi koi)	0.5000000	0.5000000	0.001474926
Urban	Bread	0.1666667	0.5000000	0.001474926
Urban	Bacon	0.5000000	0.5000000	0.001474926
Urban	Boiled fish	0.5000000	0.5000000	0.001474926
Urban	Meat	0.2500000	0.5000000	0.001474926
Urban	Tinned Meat	0.1666667	0.5000000	0.001474926
Urban	Taro	0.5000000	0.5000000	0.001474926
Urban	Mutton	0.2500000	0.5000000	0.001474926
Urban	Flour	0.5000000	0.5000000	0.001474926
Urban	Noodles	0.2500000	0.5000000	0.001474926
Urban	Tea	0.5000000	0.5000000	0.001474926
Urban	Oat porridge	0.5000000	0.5000000	0.001474926
Urban	Corned Beef	0.1666667	0.3333333	0.000983284
Urban	Salted Fish	0.3333333	0.3333333	0.000983284
Urban	Tinned food	0.1666667	0.3333333	0.000983284
Urban	Seashells	0.3333333	0.3333333	0.000983284
Urban	Taro leaves	0.1666667	0.3333333	0.000983284
Urban	Breads	0.3333333	0.3333333	0.000983284
Urban	Goose	0.1666667	0.1666667	0.000491642

GROUPING	CODE	MeanSalience	SumSalience	SmithsS
Urban	Fried Chicken	0.1666667	0.1666667	0.000491642
Urban	Coffee	0.1666667	0.1666667	0.000491642
Urban	Water Cress	0.1666667	0.1666667	0.000491642
Urban	Crab	0.1666667	0.1666667	0.000491642
Urban	Noni	0.1666667	0.1666667	0.000491642
Urban	Lamb	0.1666667	0.1666667	0.000491642
Urban	Ice-block	0.1666667	0.1666667	0.000491642
Urban	Sliced Pork	0.1666667	0.1666667	0.000491642
Urban	Pawpaw	0.1666667	0.1666667	0.000491642
Urban	Pancake	0.1666667	0.1666667	0.000491642
Urban	Seaweed	0.1666667	0.1666667	0.000491642
Urban	Clam		0.0000000	0.000000000
Urban	Lentils (dhal)		0.0000000	0.000000000

4.6 T3 rural

Saliency and frequency of mention of all 19 food items collected in the rural category, ordered by descending salience score. Italics indicate the foods that were included in the analysis.

GROUPING	CODE	MeanSalience	SumSalience	SmithsS
Rural	*Rice*	0.3928571	22.0000000	0.366666667
Rural	*Fish*	0.3065476	17.1666667	0.286111111
Rural	*Breadfruit*	0.3200000	8.0000000	0.133333333
Rural	*Babai*	0.3636364	4.0000000	0.066666667
Rural	Coconut	0.2500000	1.5000000	0.025000000
Rural	Pumpkin	0.2666667	1.3333333	0.022222222
Rural	Doughnuts	0.3333333	1.0000000	0.016666667

GROUPING	CODE	MeanSalience	SumSalience	SmithsS
Rural	Lentils (dhal)	0.5000000	1.0000000	0.016666667
Rural	Tinned food	0.2222222	0.6666667	0.011111111
Rural	Noodles	0.3333333	0.6666667	0.011111111
Rural	Banana	0.5000000	0.5000000	0.008333333
Rural	Bread	0.5000000	0.5000000	0.008333333
Rural	Tinned Fish	0.3333333	0.3333333	0.005555556
Rural	Vegetables	0.1666667	0.3333333	0.005555556
Rural	Seashells	0.3333333	0.3333333	0.005555556
Rural	Chicken	0.1666667	0.1666667	0.002777778
Rural	Salted Fish	0.1666667	0.1666667	0.002777778
Rural	Flour	0.1666667	0.1666667	0.002777778
Rural	Clam	0.1666667	0.1666667	0.002777778
Rural	Cabbage		0.0000000	0.000000000
Rural	Corned Beef		0.0000000	0.000000000
Rural	Shellfish(koikoi)		0.0000000	0.000000000
Rural	Goose		0.0000000	0.000000000
Rural	Sugar		0.0000000	0.000000000
Rural	Bacon		0.0000000	0.000000000
Rural	Raw Fish		0.0000000	0.000000000
Rural	Fried Chicken		0.0000000	0.000000000
Rural	Sausage		0.0000000	0.000000000
Rural	Boiled fish		0.0000000	0.000000000
Rural	Toddy		0.0000000	0.000000000
Rural	Coffee		0.0000000	0.000000000
Rural	Water Cress		0.0000000	0.000000000
Rural	Potatoes		0.0000000	0.000000000
Rural	Pork		0.0000000	0.000000000

GROUPING	CODE	MeanSalience	SumSalience	SmithsS
Rural	Beef Meat		0.0000000	0.000000000
Rural	Meat		0.0000000	0.000000000
Rural	Green leaves		0.0000000	0.000000000
Rural	Octopus		0.0000000	0.000000000
Rural	Crab		0.0000000	0.000000000
Rural	Cassava		0.0000000	0.000000000
Rural	Tinned Meat		0.0000000	0.000000000
Rural	Taro		0.0000000	0.000000000
Rural	Mutton		0.0000000	0.000000000
Rural	Noni		0.0000000	0.000000000
Rural	Taro leaves		0.0000000	0.000000000
Rural	Lamb		0.0000000	0.000000000
Rural	Tea		0.0000000	0.000000000
Rural	Ice-block		0.0000000	0.000000000
Rural	Sliced Pork		0.0000000	0.000000000
Rural	Breads		0.0000000	0.000000000
Rural	Pawpaw		0.0000000	0.000000000
Rural	Pancake		0.0000000	0.000000000
Rural	Oat porridge		0.0000000	0.000000000
Rural	Seaweed		0.0000000	0.000000000
